# Sense-oriented *Alu*YRa1 elements provide a lineage-specific transcription environment for polyadenylation

**DOI:** 10.1038/s41598-021-83360-4

**Published:** 2021-02-11

**Authors:** Hyeon-Mu Cho, Se-Hee Choe, Young-Hyun Kim, Hye-Ri Park, Hee-Eun Lee, Ja-Rang Lee, Sang-Je Park, Jae-Won Huh

**Affiliations:** 1 National Primate Research Center, Korea Research Institute of Bioscience and Biotechnology (KRIBB), Cheongju, Korea; 2 Department of Functional Genomics, KRIBB School of Bioscience, University of Science and Technology (UST), Daejeon, Korea; 3 Primate Resources Center, Korea Research Institute of Bioscience and Biotechnology (KRIBB), Jeongeup, Korea

**Keywords:** Evolutionary genetics, Molecular evolution

## Abstract

Transposable elements cause alternative splicing (AS) in different ways, contributing to transcript diversification. Alternative polyadenylation (APA), one of the AS events, is related to the generation of mRNA isoforms in 70% of human genes. In this study, we tried to investigate *Alu*YRa1s located at the terminal region of cynomolgus monkey genes, utilizing both computational analysis and molecular experimentation. We found that ten genes had *Alu*YRa1 at their 3′ end, and nine of these *Alu*YRa1s were sense-oriented. Furthermore, in seven genes*, Alu*YRa1s were expected to have a similar consensus sequence for polyadenylation cleavage. Additional computational analysis using the annotation files from the UCSC database showed that *Alu*YRa1 was more involved in polyadenylation than in open reading frame exon splicing. To examine the extent of *Alu*YRa1 involvement in polyadenylation, RNA-seq data from 30 normal cynomolgus monkeys were analyzed using TAPAS, a recently devised software that detects all the promising polyadenylation sites including APA sites. We observed that approximately 74% of possible polyadenylation sites in the analyzed genes were provided by sense-oriented *Alu*YRa1. In conclusion, *Alu*YRa1 is an Old-World monkey-specific TE, and its sense-oriented insertion at the 3′UTR region tends to provide a favorable environment for polyadenylation, diversifying gene transcripts.

## Introduction

Transposable elements (TEs) are repetitive movable DNA sequences on the genome^1^. They serve as driving forces contributing to genome evolution and are pathogenic elements for numerous diseases^2^. Therefore, the impact of TEs on the genome is widely studied in various areas of biological science, including fundamental and applied biology. TEs account for ~ 45% of the human genome and are divided into two major classes, namely DNA transposons and retrotransposons^1^. Unlike DNA transposons (constituting ~ 3% of the human genome), which are cut off from one genomic location and inserted into an another one, retrotransposons are transcribed into an mRNA which could be both translated and reverse transcribed, and then integrated into the new genomic site^1^. Retrotransposons have two subclasses: Long Terminal Repeat (LTR) type-like human endogenous retroviruses (HERVs, ~ 8%) and non-LTR types, such as long interspersed nuclear elements (LINEs, ~ 21%) and short interspersed nuclear elements (SINEs, ~ 13%)^3,4^.

All natural genomes are significantly influenced by these mobile elements in many different ways^5^, leading to alternative splicing (AS), a post-transcriptional process that causes alterations in the exon structure^6^. It was previously reported that > 95% of human multi-exon genes undergo AS^7,8^. AS events are usually classified into several subtypes: exon skipping (cassette exons), alternative 5′-splice site (5′-SS), alternative 3′-splice site (3′-SS), intron retention, mutually exclusive exons, alternative promotor, and alternative polyadenylation^9–12^. In mammals, > 70 and > 50% of genes are associated with alternative polyadenylation and transcription start site, respectively^13^.

Alternative polyadenylation (APA) occurs when there are multiple polyadenylation sites on a single gene transcript unit^14^. The polyadenylation mechanism is a two-step process that entails endonucleolytic cleavage at the 3′ end and subsequent synthesis of a polyadenosine (poly-A) tail, completing mature mRNA in eukaryotes^14,15^. Poly-A tail length is an essential factor for mRNA stability^14,16^, as mRNAs with short tails are vulnerable to enzymatic degradation or stored in a translationally dormant state^17^. In recent studies, the location where polyadenylation occurs, at 3′UTR or upstream of the 3′ of most exons, is proven to be crucial because it correlates with protein expression and localization^14^.

The *Alu* element, one of the primate-specific SINEs, was evolutionarily derived from 7SL RNA ~ 65 million years ago^18,19^. It is the most evolutionarily successful element, with more than 1 million copies, equivalent to 11% of the human genome^20,21^. This element is about 300 bases long, formed from two similar monomers, called “left-arm” and “right-arm”^18,21^. Antisense *Alu* in the genic region tends to provide potential splicing donor (GT) and acceptor (AG) sites, creating a new exon for the transcript^10,22^. This *Alu*-involved AS event accounts for ~ 5% of all internal alternative exons in the human genome^23^. Moreover, recent studies showed that *Alu* insertion within or near the genes in the sense orientation generates new cleavage and polyadenylation sites^24^ and establishes a dynamic polyadenylation signal (PAS) in the gene^25^. *Alu*YRa1, one of the 14 different Old-World monkey (OWM)-specific *Alu*Y subfamilies, emerged after the hominoid-OWM divergence^26^. In our brief in silico analysis, we found several genes that contain sense-oriented *Alu*YRa1 at their 3′UTR end in the cynomolgus macaque genome. We focused on macaque monkeys because they are considered a crucial animal model for biomedical research owing to their behavioral, physiological, and genetic similarities to humans^27,28^. They also have ~ 50% of TEs on the genome, similar to that in the human genome^29^.

In the present study, we aimed to analyze all *Alu*YRa1 elements in the cynomolgus macaque. Molecular experiments were performed to validate the *Alu*YRa1 insertion in cynomolgus macaques and its transcript expression, and computational analyses were carried out to investigate its characteristics. The results of this study might provide essential clues for further biomedical studies in humans.

## Results

### Computational analysis of *Alu*YRa1

**Figure 1:**
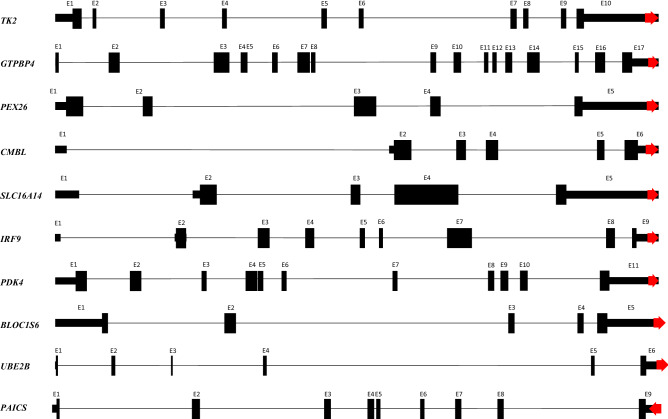
Identification of genes that had an *Alu*YRa1-derived 3′UTR-end. Ten genes were identified. Red arrows indicate *Alu*YRa1. Vertically longer boxes and shorter boxes represent ORF and UTR, respectively. This illustration is not drawn to scale. *ORF* open reading frame. *UTR* untranslated region.

Several *Alu*YRa1s, located at the 3′UTR-end of the transcripts, were identified in our brief in silico analysis on the cynomolgus monkey. Before the investigation, we computationally analyzed how many *Alu*YRa1s are located at the 3′UTR-end. First, we counted the number of genes that have a 3′UTR end located at *Alu*YRa1 via computational calculations. We found that the transcripts of seven genes (*TK2*, *PEX26*, *GTPBP4*, *IRF9*, *BLOC1S6*, *UBE2B*, *PAICS*) had 3′UTR end *Alu*YRa1 sequences. When we expanded the search to the SINE family, 72 elements were located at the 3′UTR-end of each gene; this is higher than the LINE and LTR family that had 36 and 16, respectively. Among 72 SINEs, there were 56 *Alu*s, 13 MIRs, 2 FLAMs, and 1 FRAM. Among 56 *Alu*s, 11 belonged to the *Alu*J family, 18 to the *Alu*Y family, and 27 to the *Alu*S family. To be specific, eight were *Alu*Sz, seven were *Alu*Sx1, and seven were *Alu*YRa1. *Alu*YRa1 was one of the most widely available *Alu* elements associated with transcript termination, as observed from our results. When we further considered the accession number XM, which refers to the predicted transcript, three more genes (*CMBL*, *SLC16A14*, *PDK4*) were identified to have the *Alu*YRa1 at their 3′UTR-end. To sum up, 10 *Alu*YRa1s (seven in registered genes and three in predicted genes) were located at the terminal region of the gene transcript of cynomolgus macaque (Fig. 1).

### Comparative studies on gene structure following *Alu*YRa1 insertion

**Figure 2:**
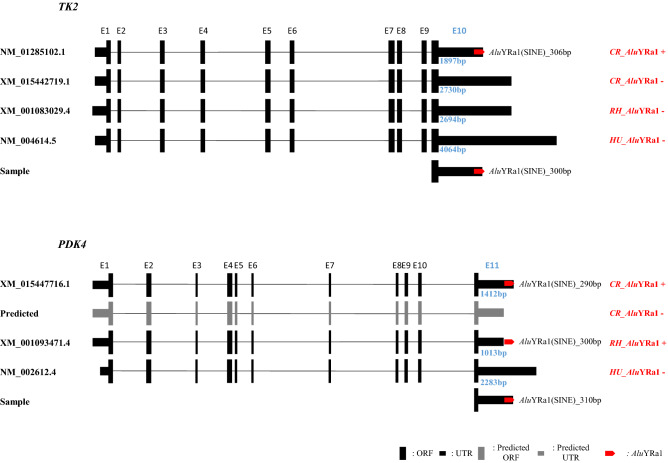
Structural analysis of *TK2 and PDK4* of cynomolgus macaque, rhesus macaque, and humans. The sense-oriented *Alu*YRa1s are located at the terminal region of the transcripts of the cynomolgus macaque. Vertically longer boxes and shorter boxes represent ORF and UTR, respectively, and the red arrow box represents *Alu*YRa1. Gray boxes represent a predicted transcript because they are not yet registered in the database. This figure is a structural illustration and is not drawn to scale. *ORF* open reading frame, *UTR* untranslated region, *CR* crab-eating monkey, *RH* rhesus monkey, *HU* human.

Ten gene structures in three genomes, namely human, rhesus macaque, and cynomolgus macaque genomes, were comparatively analyzed (Fig. 2 and Supplementary Fig. S1). As *Alu*YRa1 is Old World monkey-specific^26^, it does not exist in the human genome. We focused mostly on the structure of the 3′UTR to examine the *Alu*YRa1 insertion at the 3′UTR-end and determined the difference in the 3′UTR length between human and macaque lineage monkeys. In 8 (*TK2*, *PDK4*, *GTPBP4*, *PEX26*, *CMBL*, *BLOC1S6*, *UBE2B*, *PAICS*) out of 10 genes, the 3′UTR length of the cynomolgus macaque was shorter than that of humans (Fig. 2 and Supplementary Fig. S1). For instance, the 3′UTR length of *PDK4,* which had an *Alu*YRa1 sequence, was 1412 bp in the cynomolgus macaque but 2283 bp in humans (Fig. 2). In the case of *TK2,* which had an *Alu*YRa1, the 3′UTR length was 4064 bp in humans but 1897 bp in the cynomolgus macaque. Additionally, in four rhesus macaque genes (*PDK4*, *GTPBP4*, *CMBL*, *IRF9*) that were registered in the database, the transcript terminated at or near the *Alu*YRa1 insertion site (Fig. 2 and Supplementary Fig. S1). Meanwhile, in the case of two genes (*SLC16A14*, *IRF9*), the 3′UTR length was similar among the three species, regardless of the *Alu*YRa1 insertion.

### Evolutionary analysis of *Alu*YRa1 integration and polymorphism

**Figure 3:**
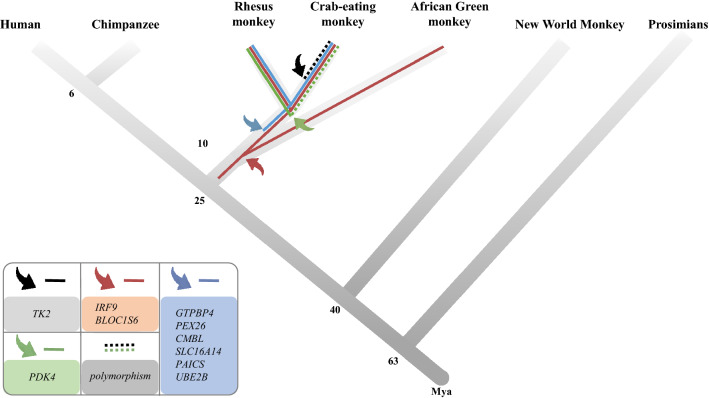
Schematic representation of the integration of *Alu*YRa1 in 10 genes during primate evolution. Bent arrows and dotted lines represent the integration of *Alu*YRa1s and polymorphism of *Alu*YRa1 insertion, respectively. Each color indicates the group of genes that contain associated *Alu*YRa1. Mya: millions of years ago.

The integration time of target *Alu*YRa1s was traced using genomic DNA samples from nine primates: hominoids (human, chimpanzee, gorilla), OWMs (rhesus monkey, cynomolgus monkey, African green monkey), New World monkeys (NWMs; marmoset, squirrel monkey) and prosimians (ring-tailed monkey). Genomic PCR and the subsequent gene cloning step were performed to confirm and analyze *Alu*YRa1 sequences. The alignment of these sequences revealed that the approximate integration time was different depending on the genes. For six genes (*GTBPB4*, *PEX26*, *CMBL*, *SLC16A14*, *PAICS*, *UBE2B*), *Alu*YRa1 was located at the terminal region of each gene in two macaques, the cynomolgus monkey, and the rhesus monkey. For two other genes (*IRF9*, *BLOC1S6*), however, the same *Alu* was located at the terminal region of the genes in two macaques and the African green monkey. This evolutionary analysis indicates that the *Alu*YRa1 in the former six genes were integrated into the 3′UTR-end of the macaque genome after it diverged from the African green monkey approximately 10 million years ago^30^. The one in the latter two genes was inserted into the 3′UTR-end between 10 and 25 million years ago after OWM diverged from hominoids (Fig. 3).

In the genomic PCR analysis, polymorphic *Alu*YRa1 was detected in two genes, *TK2* and *PDK4* (Fig. 2). For *TK2*, the first round of genomic PCR did not show any insertion of *Alu*YRa1 in the cynomolgus macaque, although the genomic sequence registered in the database had it. Therefore, we expanded the number of samples to 10 to check if *Alu*YRa1 was indeed inserted into the terminal region of the *TK2* transcript (Supplementary Fig. S2). We found that, of 10 samples, four had *Alu*YRa1-inserted allele, one had *Alu*YRa1-vacant allele, and five had both inserted and vacant alleles, which meant that the target *Alu*YRa1 in *TK2* was polymorphic. Similarly, for *PDK4*, we could not detect *Alu*YRa1-inserted allele in the first round of genomic PCR, and hence we expanded the number of samples to 10. It turned out that two samples had a vacant allele, three had an inserted allele, and five had both alleles. These also represented the polymorphism of target *Alu*YRa1 in *PDK4.* The results indicate that *Alu*YRa1 integrated into *TK2* after it was diverged from the rhesus macaque and integrated into *PDK4* before that event (Fig. 3).

### Expression pattern of sense-oriented 3′UTR-end *Alu*YRa1

**Figure 4:**
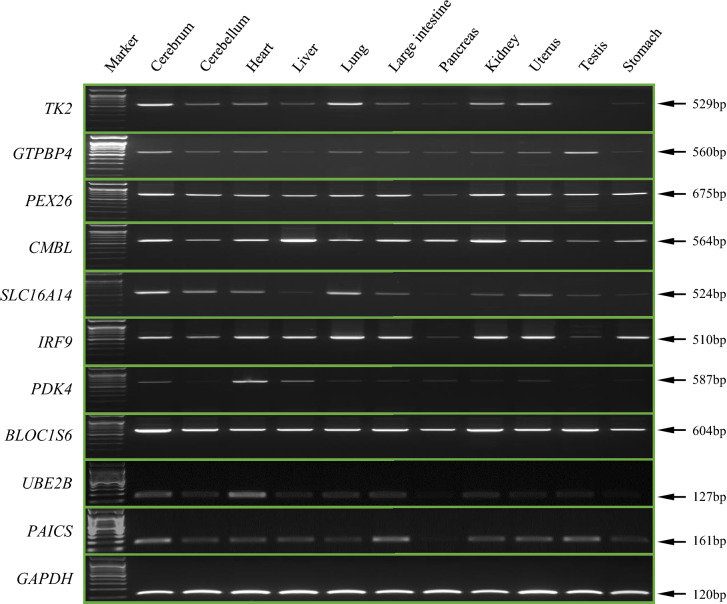
RT-PCR amplification for expression pattern analysis of 10 genes in the crab-eating monkey. *TK2* (529 bp), *GTPBP4* (560 bp), *PEX26* (675 bp), *CMBL* (564 bp)*, SLC16A14* (524 bp), *IRF9* (510 bp), *PDK4* (587 bp), *BLOC1S6* (604 bp), *UBE2B* (127 bp), *PAICS* (161 bp) were amplified with a transcript-specific primer set (Supplementary Table S3 and Supplementary Fig. S2). *GAPDH* (120 bp) indicates positive control. Full-length gels are presented in Supplementary Fig. S6.

Reverse transcriptase PCR (RT-PCR) amplification was conducted to check if target *Alu*YRa1 was expressed as part of the transcript, and also observe its expression patterns. cDNA from 11 tissues (cerebrum, cerebellum, heart, liver, lung, large intestine, pancreas, kidney, uterus, testis, stomach), extracted from cynomolgus monkey, were used for RT-PCR. The results showed that the target *Alu*YRa1 was ubiquitously expressed in various tissues of all analyzed genes (Fig. 4). The patterns of expression level specifically displayed that the targets of three genes (*PEX26*, *CMBL*, *BLOC1S6*) were strongly expressed in all eleven tissues, and the targets of all analyzed ten genes were expressed in cerebrum and heart.

### Structural analysis of sense-oriented *Alu*YRa1s at the 3′UTR-end

**Figure 5:**
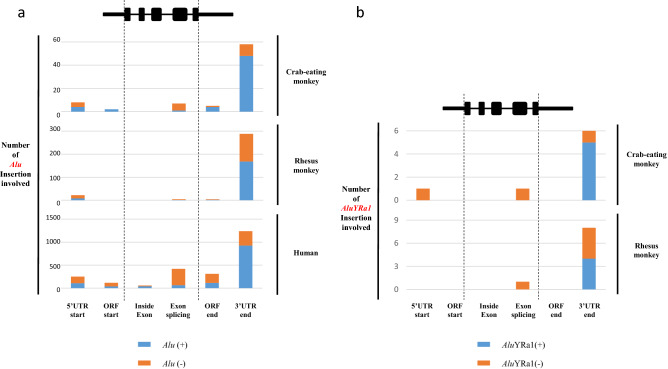
Calculation of repeat element position in three genomes: cynomolgus macaque, rhesus macaque, and human. A self-devised locator was utilized to provide the output data in a certain format, and the graphs are based on results of the statistical sorting of the output file for cynomolgus macaque, rhesus macaque, and humans. (**a**) *Alu* detailed position information. (**b**) *Alu*YRa1 position information. Blue and orange bars represent the inserted repeat element in sense and antisense orientation, respectively.

To clarify the pattern by which *Alu*YRa1 was integrated into the genome, we created the Python script that we named it a locater, which indicated where all repeated elements were located in the reference gene sequence. We ran the locator and obtained the annotated file as an output. We first sorted the information on all *Alu*s in the output file before specific *Alu*YRa1 analysis. It revealed that *Alu*s were located at 3′ UTR-end more frequently than other regions (5′UTR start, ORF start, inside exon, exon splicing, and ORF end) in all the three species (Fig. 5a). Besides, sense-oriented *Alu*s were almost three times more frequently observed than antisense-oriented ones in the gene termination region of cynomolgus monkeys and humans (Fig. 5a). On the contrary, antisense-oriented *Alu*s were found in the exon splicing junction of human reference genes six times more than the sense-oriented ones, and similar patterns were observed in cynomolgus and rhesus monkeys (Fig. 5a). When we focused on specific *Alu* subfamilies, *Alu*J, *Alu*S, and *Alu*Y, we could see similar patterns, and the *Alu*S family was proportionally the most abundant at the 3′UTR-end, especially in humans (Supplementary Fig. S3). Next, we did the same sorting for *Alu*YRa1, and we realized that *Alu*YRa1s were located at the 3′UTR-end more than the exon splicing junction, as observed in the patterns of *Alu*s (Fig. 5b). The ratio of sense- and antisense-oriented *Alu*YRa1 was also similar to that of *Alu*s (Fig. 5b).

**Figure 6:**
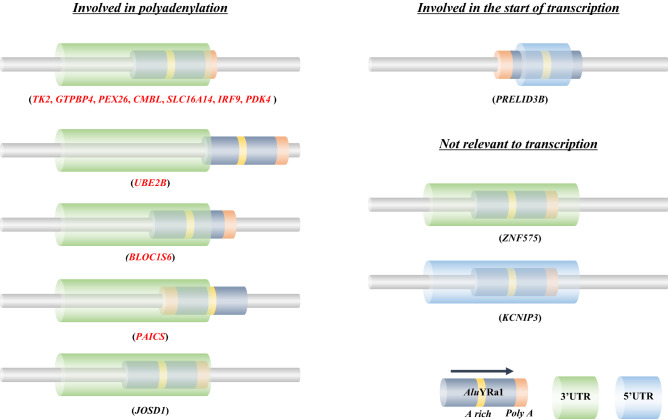
Structural analysis of sense-oriented *Alu*YRa1 insertion in cynomolgus macaque. Fourteen genes located at the UTR of the transcript were identified. These *Alu*YRa1s were classified into three categories based on their characteristics. Green and blue horizontal cylinders represent 3′UTR and 5′UTR, respectively. Black horizontal cylinder with a yellow one (A-rich region) and an orange one (poly-A tail region) represent *Alu*YRa1. Black arrow indicates the insertion direction of *Alu*YRa1. UTR; untranslated region.

We subsequently analyzed the detailed structure of *Alu*YRa1-inserted sections in the genes using the information from the output file described above. Therefore, 14 genes were identified to have *Alu*YRa1 at their 3′ and 5′ UTR regions (Fig. 6). Two genes (*ZNF575*, *KCNIP3*) had *Alu*YRa1 in the middle UTR, which was not relevant to AS events during transcription. In the case of *PRELID3B*, antisense-oriented *Alu*YRa1 was intriguingly involved in the generation of both ends of exon number 1, which was associated with the start of transcription. In *JOSD1*, the *Alu*YRa1 sequence was near the 3′UTR termination region, but it did not overlap. Finally, the remaining 10 genes had their *Alu*YRa1 sequences overlapped with the 3′UTR-end (Fig. 6). Among these 10 genes, the 3′UTR-ends of *UBE2B*, *BLOC1S6,* and *PAICS* were located on the right-arm, A-rich region, and the left-arm of *Alu*YRa1, respectively. In the case of seven genes (*TK2*, *GTPBP4*, *PEX26*, *CMBL*, *SLC16A14*, *IRF9*, *PDK4*), sense-oriented *Alu*YRa1 was located at the end of the gene transcript sequence, and precisely that location was at or near the poly-A tail of *Alu*YRa1 (Fig. 6). They had a similar cleavage sequence (CA) based on the analyzed sequence^31,32^ (Supplementary Tables S1 and S2)^33^.

### *Alu*YRa1 is the element that gives rise to possible APA sites

**Figure 7:**
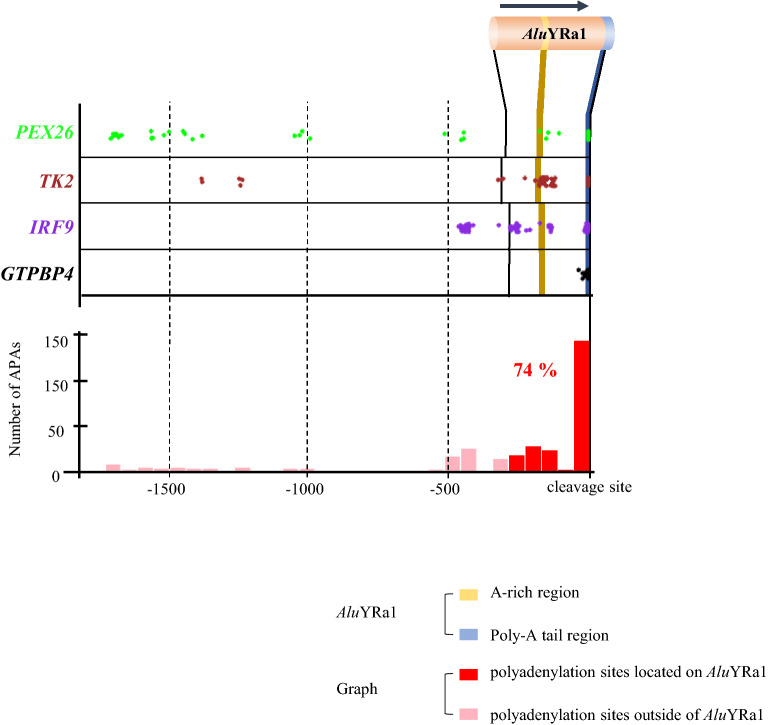
TAPAS analysis. The TAPAS software run on 30 cynomolgus macaques resulted in a certain output file, and we statistically analyzed this output file. Dots represent APA sites. Pink bar indicates the accumulation of the dots (APA sites) above, and the red bar represents the same, but the total red bars represent the total APA sites that are located on the *Alu*YRa1 sequence. The total ratio is 74%. APA; alternative polyadenylation.

*Alu*YRa1 is located at the terminal region of 10 gene transcripts of cynomolgus monkey, as mentioned above, and it tended to be overlapped with the sequences that were associated with the polyadenylation cleavage site. Therefore, we were curious to know how closely *Alu*YRa1 was related to the terminal sequence of the transcript. To do this, we used TAPAS software which is the tool for detecting such alternative (or all) polyadenylation sites within a gene from RNAseq data^34^. We downloaded relevant reference annotation file (refFlat.txt) and 30 individuals of RNA sequencing data as input files to run TAPAS. We finally achieved reliable output files that showed information on the polyadenylation site of each gene. Of the seven genes that had the sense-oriented *Alu*YRa1 in which its poly-A region overlapped with the 3′UTR-end, 4 (*PEX26*, *TK2*, *IRF9*, *GTPBP4*) were included in the reference annotation file (refFlat.txt), which means they were qualified for TAPAS analysis of this study. The results data revealed that approximately 74% of polyadenylation site including APA sites were located on the sense-oriented *Alu*YRa1 sequences (Fig. 7). To be specific, 40% of them were located on the poly-A tail and another 34% were on the A rich region of the sense-oriented *Alu*YRa1 (Fig. 7).

## Discussion

### Sense-oriented *Alu*YRa1 offers a proper environment for polyadenylation

*Alu* sequence coupled with polyadenylation has rarely been studied, although their correlation has been reported before. In 2009, Clen et al. analyzed the impact of the *Alu* element on polyadenylation in humans and mice and revealed that the *Alu* sequence often provides the gene with a unique PAS that prompts the cleavage of the 3′UTR end^25^. They identified putative poly-A sites based on Expressed Sequence Tag (EST)/cDNA information^25^. Since now we have more reliable reference gene transcript sequences over various species, which are registered at specific databases, we mainly focused on macaque monkeys. The computational analysis of the cynomolgus monkey genes along with the experimental validation in this study revealed that *Alu* elements were the majority of TEs that were located mostly at the terminal region of the reference gene transcript compared to the other TEs. *Alu*YRa1 was the second largest element among these *Alu*s. Therefore, we would insist that *Alu* elements were the most influential TEs on polyadenylation than any other TEs in the cynomolgus monkey and *Alu*YRa1 was also one of the most important elements. In addition, we found that more than 80% of both 3′UTR-end located *Alu*s and *Alu*YRa1 had the same orientation as the genes they were inserted into. This result indicated the possible correlation between sense-oriented *Alu*s and more powerful PAS or sensitive cleavage sites for the mRNA sequence termination mechanism.

The phenomenon of *Alu*YRa1 insertion providing different polyadenylation cleavage sites depending on the species could have an impact on the species-specific 3′UTR length. In 2012, Clen et al. proposed that 3′UTR extension is correlated with organismal complexity in animal evolution because a longer 3′UTR contains more putative targets for microRNAs, which is related to morphological complexity^35,36^. In the current study, we identified eight genes in the cynomolgus monkey that underwent 3′UTR shortening, compared to humans, via *Alu*YRa1 insertion. Therefore, we propose that *Alu*YRa1 might play evolutionary roles between humans and cynomolgus macaques in terms of different polyadenylation patterns.

### Sense-oriented *Alu*YRa1 is more associated with polyadenylation than exon splicing

In 2015, Tajnik et al. reported that intergenic *Alu* exonization contributes to both AS and polyadenylation in the upstream genes^37^. The tendency of *Alu* insertion in the antisense orientation, providing potential 5′SS or 3′SS, has been studied more than the alternative polyadenylation of the *Alu* sequence^38^. This is because the study of the exon splicing mechanism related to the diseases is essential, and the research protocol for exon splicing is simple. However, polyadenylation study associated with the *Alu* element is an increasing concern among genome biologists. According to our results, *Alu*s were located at the 3′UTR-end more than the exon splicing junction, implying that *Alu* insertion was more influential in the polyadenylation than exon splicing. We also verified that *Alu*s prompting polyadenylation tended to be more sense-oriented, whereas those involved in exon splicing were antisense-oriented.

With these results, we could predict that *Alu*s were possible causative elements for polyadenylation. To make our results more reliable, we conducted RNA transcriptome sequencing on 30 cynomolgus monkeys and checked the expression patterns of their 3′UTR-end using the TAPAS software. The result from the run showed that 74% of predicted polyadenylation sites and APA sites were located at the inserted *Alu*YRa1 sequence, showing that *Alu*YRa1 elements were correlated with polyadenylation phenomenon including APA. Hence, we proposed that *Alu*YRa1 insertion at the 3′UTR generated various APA sites contributing to the diversification of transcripts. The computational analysis in this study also represented that the same phenomenon was likely to occur with many other *Alu* elements because our results showed that numerous *Alu*s were located at the 3′UTR-end.

3′UTRs have been reported to adjust mRNA stability through the regulation of gene expression via RNA binding protein, determining the protein levels^36,39^. Moreover, the localization element located on the 3′UTRs mediates mRNA localization for translation^40^. In the same manner, various transcripts with different 3′UTR lengths generated by *Alu*YRa1 insertion could alter gene expression and mRNA localization in a tissue-specific or species-specific way. Therefore, it is possible to conclude that these changes affect the phenotypic function of the organisms or lead to diseases such as cancer^41^.

## Conclusion

From the above pieces of evidence, *Alu*YRa1, an Old-World monkey-specific TE, tends to provide appropriate conditions for polyadenylation when it is sense-oriented, contributing to the diversification of gene transcripts. Further computational analyses also indicate that *Alu*s tends to follow the same phenomenon as *Alu*YRa1. The results of this study, such as *Alu*-mediated polyadenylation pattern and cynomolgus macaque-specific *Alu*YRa1 polymorphism in the several genes, might be a valuable source for future non-human primate research. Further, an in-depth analysis of TE distribution patterns between non-human primates and humans would lead to therapeutic advances in biomedical research and evolutionary understanding of primate radiation.

## Materials and methods

### Ethics declarations

Human and rhesus monkey samples were purchased from Clontech Laboratories, Inc. Crab-eating monkey, chimpanzee, gorilla and African green monkey samples were provided by the NPRC of the KRIBB. All procedures, including animal sample preparation and the study design, were performed following the Guidelines of the Institutional Animal Care and Use Committee of the KRIBB (Approval No. KRIBB-AEC-20080).

### Genomic DNA and total RNA samples

All the following DNA samples were provided by the late Prof. Osamu Takenaka from the Primate Research Institute of the Kyoto University of Japan and the National Primate Research Center (NPRC) of Korea. (1) Hominoids: HU, humans (*Homo sapiens*), CH, chimpanzees (*Pan troglodytes*), and GO, gorilla (*Gorilla gorilla*); (2) OWMs: RH, rhesus monkeys (*Macaca mulatta*), CR, crab-eating monkeys (*Macaca fascicularis*), AGM, African green monkeys (*Chlorocebus aethiops*); (3) NWMs: MAR, marmosets (*Callithrix jacchus*) and SQ, squirrel monkeys (*Saimiri sciureus*); (4) prosimians: RL, ring-tailed lemurs (*Lemur catta*).

Total RNA samples are from the tissues of adult crab-eating monkey (*Macaca fascicularis*; cerebrum, cerebellum, heart, liver, lung, large intestine, pancreas, kidney uterus, testis, and stomach) that originated from Vietnam and imported from China under a Convention on International Trade in Endangered Species of Wild Fauna and Flora (CITES) permit. These total RNA samples were extracted using the RNeasy Plus Mini kit (Qiagen, Hilden, Germany) according to the manufacturer’s instructions. DNA contamination was prevented by using the genomic eliminator column and RNase-free DNase (Qiagen, Hilden, Germany). The RNA concentration and purity (A260/A280) were measured with the ND-1000 spectrophotometer. The integrity of the total RNA was confirmed by agarose gel electrophoresis. Only about 500 ng of total RNA samples proceeded to the complementary DNA synthesis step. GoScript Reverse Transcription System (Promega, Madison, Wisconsin, USA) was used for the synthesis according to the manufacturer’s instructions.

### PCR and RT-PCR amplification

The target *Alu*YRa1 insertion sites at the terminal region of 14 gene transcripts (*TK2*, *GTPBP4*, *PEX26*, *CMBL*, *SLC16A14, IRF9*, *PDK4*, *BLOC1S6*, *ZNF575*, *PAICS*, *UBE2B*, *JOSD1*, *KCNIP3*, *PRELID3B*) were identified using genomic PCR, which was conducted using the ExPrime Taq Premix (GenetBio, Daejeon, Korea). The PCR conditions were: 30–35 cycles of 30 s at 94 °C, 30 s at 58 °C, 30 s at 72 °C. The number of cycles varied depending on the genes. For *BLOC1S6*, however, genomic DNA samples of nine primates were divided into two groups as an exception because of the different annealing temperatures in marmoset, squirrel monkey, and ring-tailed lemur. Therefore, two different primer pairs were designed: one for humans, chimpanzees, gorillas, rhesus monkeys, crab-eating monkeys, and African green monkeys, and another for marmosets, squirrel monkeys and ring-tailed lemurs (Supplementary Table S3). The genomic PCR conditions for the former group were: 35 cycles of 30 s at 94 °C, 30 s at 57 °C, and 30 s at 72 °C. PCR conditions for the latter group were: 35 cycles of 30 s at 94 °C, 30 s at 55 °C, and 60 s at 72 °C.

Ten genes (*TK2*, *GTPBP4*, *PEX26*, *CMBL*, *SLC16A14*, *IRF9*, *PDK4*, *BLOC1S6*, *UBE2B*, *PAIC*S) were analyzed by RT-PCR using the ExPrime Taq Premix (GenetBio, Daejeon, Korea), and those carried out as following conditions: 30–35 cycles of 30 s at 94 °C for, 30 s at 55–58 °C, and 30 s at 72 °C. The number of cycles and annealing temperature varied depending on different genes. Specific RT-PCR primer pairs were also designed differently depending on the genes (Supplementary Table S3). The GAPDH gene, a standard control, was analyzed using specific primer pairs (S: 5′-GAA ATC CCA TCA CCA TCT TCC AGG-3′, AS: 5′-GAG CCC CAG CCT TCT CCA TG-3).

### Molecular cloning and sequencing procedures

PCR products were separated on 1.2–1.5% agarose gels and purified using the Gel SV Extraction kit (GeneAll, Seoul, Korea). The purified DNA was ligated into a T&A Cloning Vector (RBC Bioscience), then transformed into ECOS 101 (Yeastern Biotech, New Taipei City, Taipei) competent cells (strain: DH5α), which were then grown on agar plates containing 100 µg/ml of ampicillin. The cloned vectors were isolated using the Hybrid-Q Plasmid Rapidprep kit (GeneAll, Seoul, Korea). PCR product sequencing was performed by a commercial sequencing company (Macrogen Inc, Seoul, Korea).

### 3′UTR-end-located TE analysis

We performed basic Python coding to build a locater that showed the specific location of repeated sequences in certain organisms. The run of this program yields detailed information about the location pattern, such as the exon number, 5′UTR or 3′UTR region, and the direction (Supplementary Fig. S4). To run the locator, we downloaded two types of input files from the UCSC database. The first input file (ftp://hgdownload.soe.ucsc.edu/goldenPath/macFas5/database/refGene.txt) was the reference gene annotation file that contained all the position information on transcript-start and -end, exon-start and -end, the number of exons, and their direction. The second input file (ftp://hgdownload.soe.ucsc.edu/goldenPath/macFas5/database/rmsk.txt) was the repeat masking information file that contained the genomic position of the repeated sequence, its name, and its direction. We ran this analysis with the two input files of the cynomolgus macaque, rhesus macaque, and humans. We sorted the output file of these 3 species to obtain all *Alu*s or *Alu*YRa1s that were located at the end of the registered gene transcript. This sorting was performed using Awk languages in Linux operating system.

### TAPAS analysis

TAPAS, a recently devised software, enabled us to detect which is the tool for detecting such alternative (or all) polyadenylation sites within a gene from RNAseq data^34^. To run TAPAS, two types of file are required; one reference annotation file (ftp://hgdownload.soe.ucsc.edu/goldenPath/macFas5/database/refFlat.txt), which was downloaded from the UCSC database, and another one called the read coverage file that was generated from the BAM file using samtools (the program that utilizes SAM file) command upon the Linux operating system. The BAM file (.bam), which contained information about the read sequence, was generated from the RNA sequencing data (unpublished) of 30 cynomolgus macaques’ blood samples (Supplementary Fig. S5)^42^. These healthy macaques were from the NPRC of the KRIBB.

The reference annotation file and read coverage file were used as input files for TAPAS analyses. The output file consisted of six columns: gene name, chromosome name, the strand of the gene, detected APA sites, the abundance of those APA sites, and read count^34^. We sorted and tried to extract the information from the output file for the 10 genes that had 3′UTR-end *Alu*YRa1s, but we could extract information on only seven genes because of the absence of information in the reference gene file for the remaining 3 genes (*CMBL*, *SLC16A14*, *PDK4*). Another group of three genes (*BLOC1S6*, *UBE2B*, *PAICS*) was also excluded from the TAPAS analysis because of their different direction or inappropriate gene structure. Therefore, we extracted APA information for four genes (*TK2*, *GTPBP4*, *PEX26*, *IRF9*) from the TAPAS output file and conducted the statistical analysis.

## Supplementary information

Supplementary Information
