# Supplementary material for: Sense-oriented AluYRa1 elements provide a lineage-specific transcription environment for polyadenylation
Source: Sci Rep. 2021 Feb 11;11:3665. doi: 10.1038/s41598-021-83360-4 (PMC7878741; doi:10.1038/s41598-021-83360-4)
Supplement: Supplementary file 1 — Supplementary Information [file 41598_2021_83360_MOESM1_ESM.pdf]

Sense-oriented *Alu*YRa1 elements provide a lineage-specific transcription environment for polyadenylation

Hyeon-Mu Cho[1,2], Se-Hee Choe[1,2], Young-Hyun Kim[1,2], Hye-Ri Park[1,2], Hee-Eun Lee [1], Ja-Rang Lee[3], Sang-Je Park[1,*], and Jae-Won Huh[1,2,*]

[1] National Primate Research Center, Korea Research Institute of Bioscience and Biotechnology (KRIBB), Cheongju, Korea

[2] Department of Functional Genomics, KRIBB School of Bioscience, Korea University of Science & Technology (UST), Daejeon, Korea

[3] Primate Resources Center, Korea Research Institute of Bioscience and Biotechnology (KRIBB), Jeongeup, Korea

* Correspondence

Corresponding Authors: parksj@kribb.re.kr, huhjw@kribb.re.kr

Supplementary Tables

Supplementary Table S1.

GENE		*Alu*YRa1-overlapped region with 3'UTR-end	*Alu*YRa1 direction	30nt upstream sequence of cleavage site
1	*TK2*	poly a tail	sense	GCCTGGGCGACAAAGCGAGACTCCGTCTCA
2	*CMBL*	poly a tail	sense	GCCTGGGCAACAGAGGGAGACTCCGTCTCA
3	*SLC16A14*	poly a tail	sense	GCCTGGGCGACAGAGCGAGACTCCGTCTCA
4	*PDK4*	poly a tail	sense	GCCCGGGCGACAGAGCGAGACTCCGTCTCA
5	*PEX26*	poly a tail	sense	GCCTGGGCGACAG - - CGAGACTCCGTCTCA
6	*GTPBP4*	poly a tail	sense	GCCTGGGCCACAGAGCAAGACTCCGTCTCA
7	*IRF9*	poly a tail	sense	GCCTGGGCGACAGAGCGAGACTCCATCTCA
8	*BLOC1S6*	right arm	sense	AGGCTGAGGCAGGAGAATGGCGTAAACCCG
9	*UBE2B*	left arm	sense	AACTCTGTATTTAGGCCATTTGTTACAGTT
10	*PAICS*	A-rich	anti	ACCGCGCCCGGCTAGTTTTTTTTTTTTTTT

Supplementary Table S1. 3'UTR-end sequence analysis.

In seven genes (*TK2*, *GTPBP4*, *PEX26*, *CMBL*, *SLC16A14*, *PDK4*, *IRF9*), the 3'UTR-end and poly tail region of *Alu*YRa1s were overlapped. Therefore, we checked the sequence of their terminal regions, and we found similar sequences for transcript cleavage sites. CAs, the end sequence of the seven genes are assumed to be the cleavage site.

Supplementary Table S2.

poly A signal motif	CountP	E-value	poly A signal motif	CountP	E-value
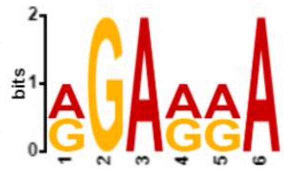	20.01	6.70E-48	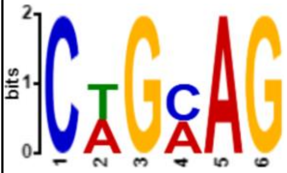	7.75	1.60E-07
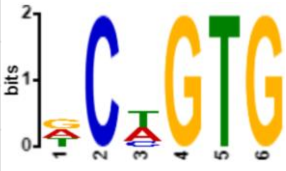	13.39	1.70E-32	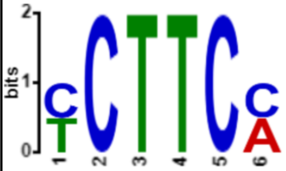	7.46	6.60E-14
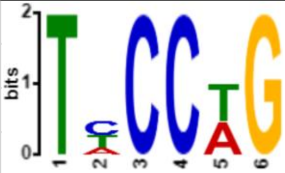	12.78	6.80E-34	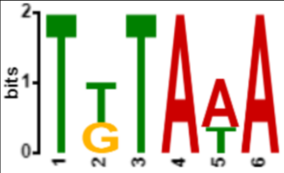	7.35	9.50E-27
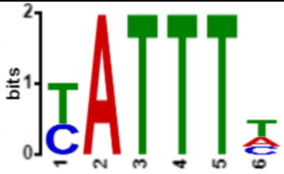	12	6.60E-48	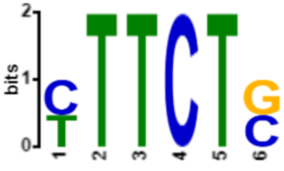	6.38	1.10E-09
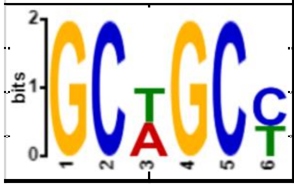	10.01	7.50E-17	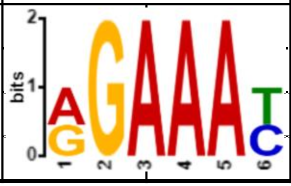	5.08	6.50E-12

Supplementary Table S2. Predicted poly A hexamer signal on cynomolgus macaque genome.

The logos, CountP and E-value of poly A singal motif are downloaded from Animal-APAdb (http://gong_lab.hzau.edu.cn/Animal-APAdb/motif/motif.html). The database informed that these motifs are generated by MEME Suite program (http://meme-suite.org). 87 tissue samples of cynomolgus macaque were used for results of the database. CountP is the percentage of sequences matching the motif. E-value represents the statistical significance of the motif.

Supplementary Table S3.

GENE	PRIMER	SEQUENCE		PRODUCT SIZE (bp)
TK2	Genomic Primer	Forward	5' - TTCCCGGTAGCAAAGGTTCT - 3'	675
		Reverse	5' - ACCCTGGGAAGGATTAAGGC - 3'	
	RT Primer	Forward	5' - CCTCAAGCCATAGAGCCTGA - 3'	529
		Reverse	5' - CCAGGATGGTCTCGATCTCC - 3'	
GTPBP4	Genomic Primer	Forward	5' - TCTGGGAAGAGGAAAGCTGG - 3'	973
		Reverse	5' - CCATATTTAATTGCCTAATGCCT - 3'	
	RT Primer	Forward	5' - CACTTGTTGCTTTGCCGAAA - 3'	560
		Reverse	5' - TTTACGCCATTCTCCTGCCT - 3'	
PEX26	Genomic Primer	Forward	5' - TGCTAGCTTTTCCTTGGTACA - 3'	452
		Reverse	5' - GCTTCCTACAGACTTGAACCG - 3'	
	RT Primer	Forward	5' - GCTCTTTCACCAGGTTCTGC - 3'	675
		Reverse	5' - TTCACCGTGTTAGCCAGGAT - 3'	
CMBL	Genomic Primer	Forward	5' - TGTCAGCTGGTCCTTGAACA - 3'	1374
		Reverse	5' - GACTCATGGGTTCGTGCATC - 3'	
	Genomic Primer (nested)	Forward	5' - GACTCATGGGTTCGTGCATC- 3'	669
		Reverse	5' - CAGTCTCCCAAGTAGCTGGG- 3'	
	RT Primer	Forward	5' - GACTCATGGGTTCGTGCATC - 3'	564
		Reverse	5' - AATGGCCAGATCTCAGCTCA - 3'	
SLC16A14	Genomic Primer	Forward	5' - TATTCTCTTCGAGGCCAACA - 3'	1041
		Reverse	5' - TTGCATGTCATATTCCATTTGG - 3'	
	RT Primer	Forward	5' - GGCAATGCTCCCTCCTCTAT - 3'	524
		Reverse	5' - TTCACCGTGTTAGCCAGGAT - 3'	

GENE	PRIMER	SEQUENCE		PRODUCT SIZE (bp)
IRF9	Genomic Primer	Forward	5' - CAAATGGTGTGAACCCAGGG - 3'	566
		Reverse	5' - AGAACCTGTGGGCAGAGGA - 3'	
	RT Primer	Forward	5' - CCCAGCCATACTCCACAGAA - 3'	510
		Reverse	5' - CCAGGATGGTCTCGATCTCC - 3'	
PDK4	Genomic Primer	Forward	5' - AGAATCTCTTACGTCCAGGCT - 3'	490
		Reverse	5' - AGGCATCCATTTTCTAGGCTT - 3'	
	RT Primer	Forward	5' - TTCAAGGTTGCAGTGAAGGC - 3'	587
		Reverse	5' - GTTTACGCCATTCTCCTGCC - 3'	
BLOC1S6	Genomic Primer (HU,CH,GO,RH,CR,AGM)	Forward	5' - TAAAGCTTGGTGGAGGTGGT - 3'	845
		Reverse	5' - ACGTATCATGCTGCCTCTTT - 3'	
	Genomic Primer (SQ,MA,LE)	Forward	5' - AAGTTTGGTGGAGGTGGT - 3'	464
		Reverse	5' - ATCTATAAAGTGGGTAGTTGGT - 3'	
	RT Primer	Forward	5' - CCCCTGGAAAAGAGGTGAGT - 3'	604
		Reverse	5' - CCAGGATGGTCTCGATCTCC - 3'	
UBE2B	Genomic Primer	Forward	5' - CATAACTGGTTTGGTTCTGTCC - 3'	464
		Reverse	5' - CAAACAGCAAAACAAGTCGCA - 3'	
	RT Primer	Forward	5' - CTGTCCAACTCTGTATTTAGGCC - 3'	127
		Reverse	5' - CGTCTCAGCCTCCCAAAGT - 3'	
PAICS	Genomic Primer	Forward	5' - TGAAGCAGGCTGACAAGAAA - 3'	600
		Reverse	5' - AGTGACCACCAATACTTAAGGAA - 3'	
	RT Primer	Forward	5' - CAGCCAGCCTTTATCACTCC - 3'	161
		Reverse	5' - AGTCCCAGCTACTCCAGAGG - 3'	

Supplementary Table S3. Primer list for genomic PCR and RT-PCR.

Supplementary Figures

Supplementary Figure S1.

Supplementary Figure S1. Gene structural analysis (Fig. 2) of the cynomolgus macaque, rhesus macaque, and humans.

Expanded to eight more genes (*GTPBP4*, *PEX26*, *CMBL*, *SLC16A14*, *IRF9*, *BLOC1S6*, *UBE2B*, *PAICS*) because these genes also had *Alu*YRa1 at their 3'UTR-ends. Vertically longer boxes and shorter boxes represent ORF and UTR, respectively, and the red arrow box represents *Alu*YRa1. This figure is not drawn to scale. ORF, open reading frame; UTR, untranslated region; CR, crab-eating monkey; RH, rhesus monkey; HU, human.

Supplementary Figure S1.

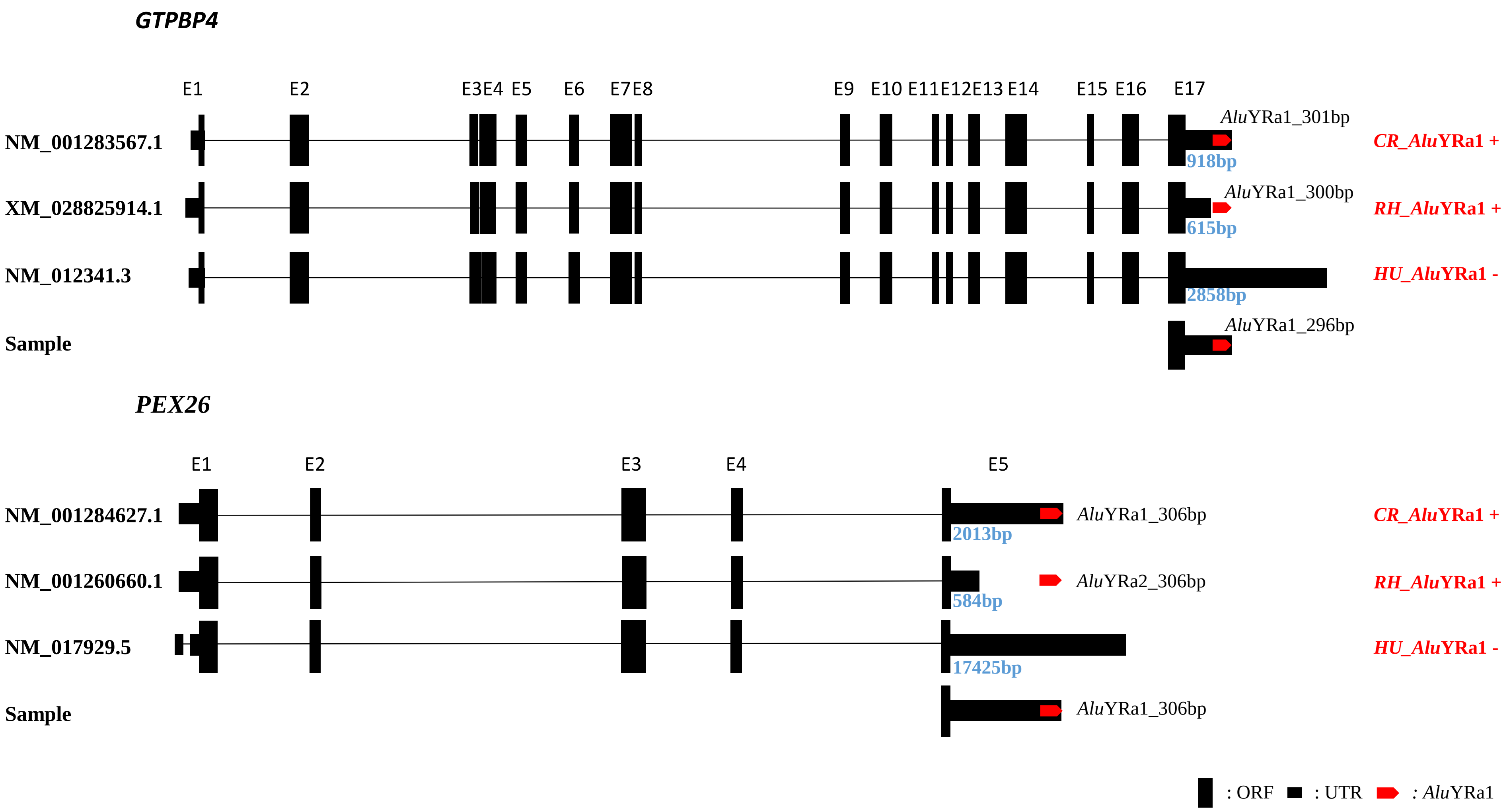

Supplementary Figure S1.

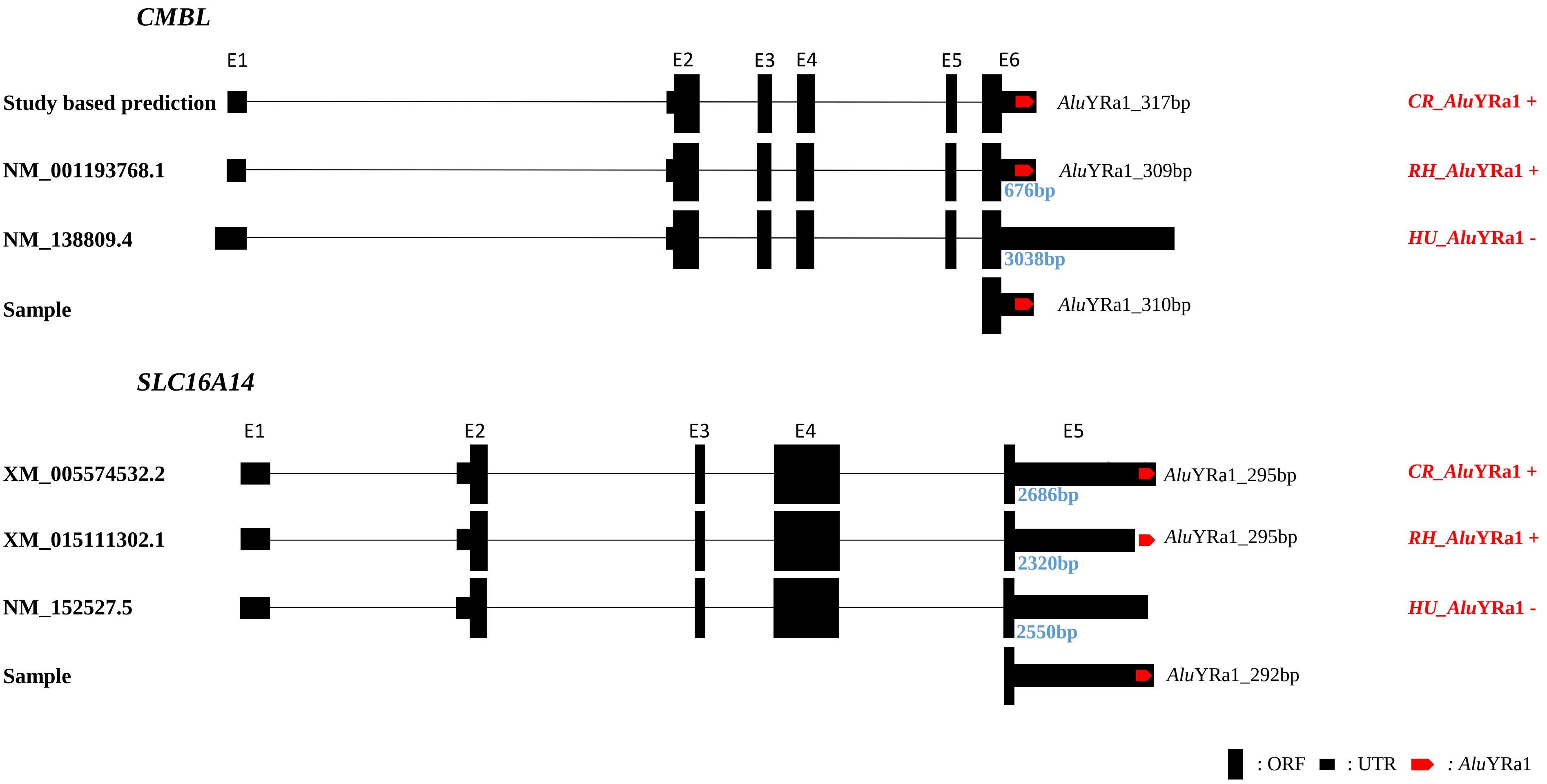

Supplementary Figure S1.

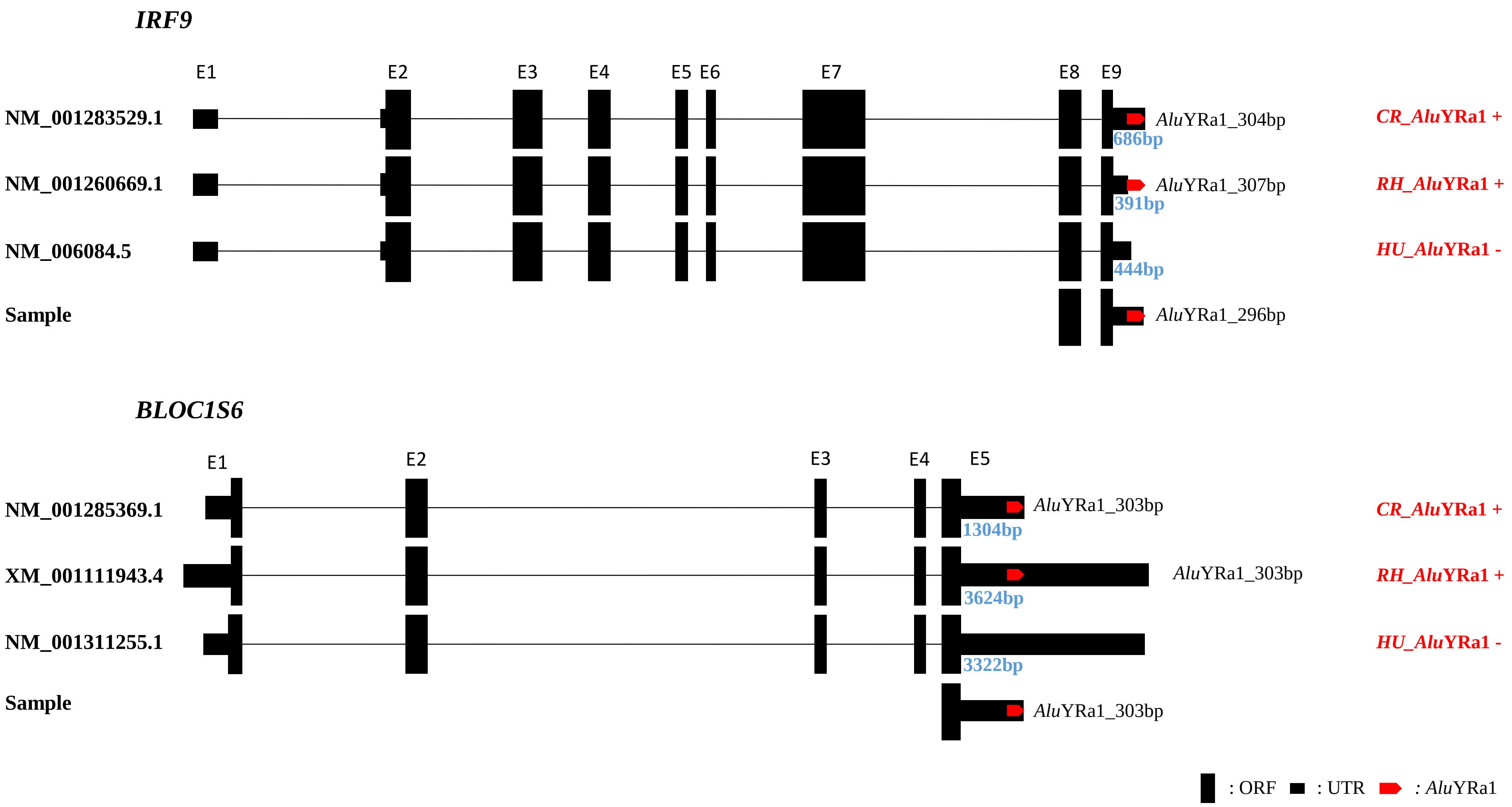

Supplementary Figure S1.

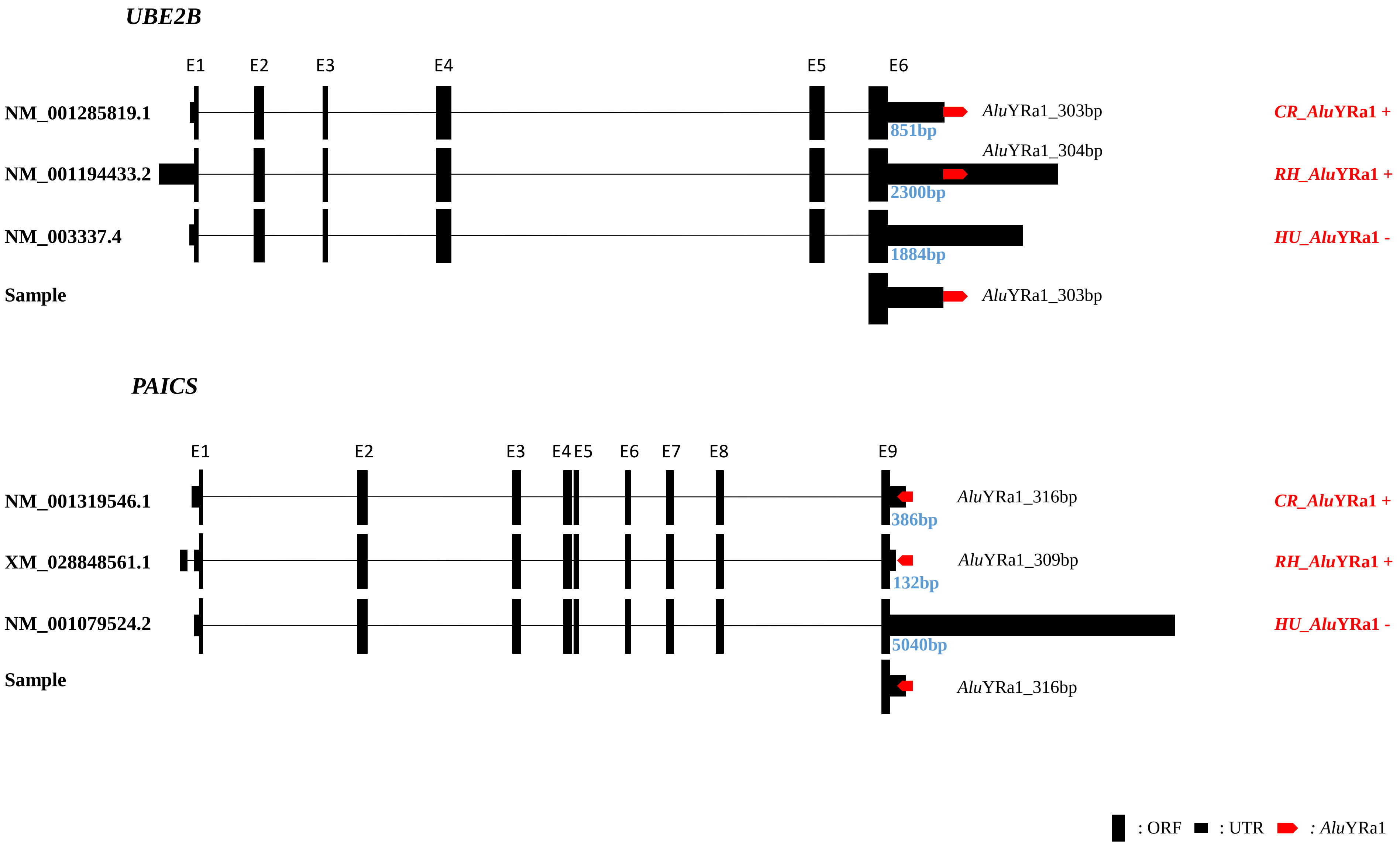

Supplementary Figure S2.

Supplementary Figure S2. Genomic DNA PCR analysis and multiple sequence alignment of the integrated *Alu*YRa1.

Ten genes (*TK2, GTPBP4, PEX26, CMBL, SLC16A14, PDK4, PAICS, UBE2B, IRF9, BLOC1S6*) in nine species. The species analyzed were: HU, human; CH, chimpanzee; GO, gorilla; RH, rhesus monkey; CR, crab-eating monkey; AGM, African green monkey; MA, marmoset; SQ, squirrel monkey; and LE, ring-tailed monkey. Yellow arrow box represents *Alu*YRa1, whereas the black arrow box represents different *Alu*s. Blue and orange arrows represent RT-PCR primer and genomic PCR primer, respectively. Blue and orange boxes also represent RT-PCR and genomic PCR products, respectively. TSD is the target site duplication. Full-length gels are presented in Supplementary Figure S7.

Supplementary Figure S2.

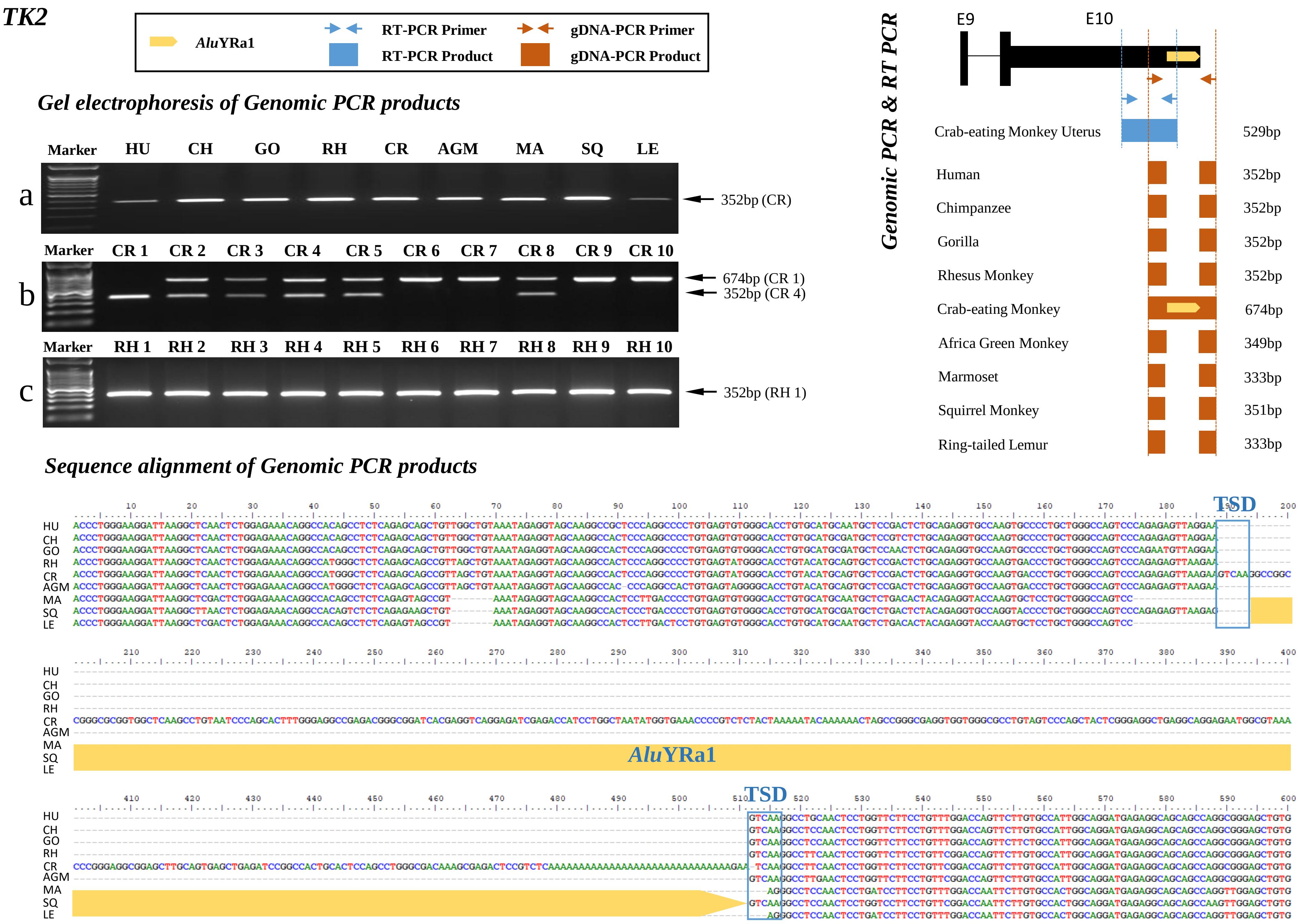

Supplementary Figure S2.

GTPBP4

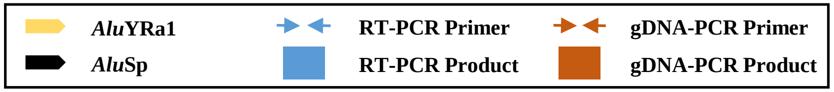

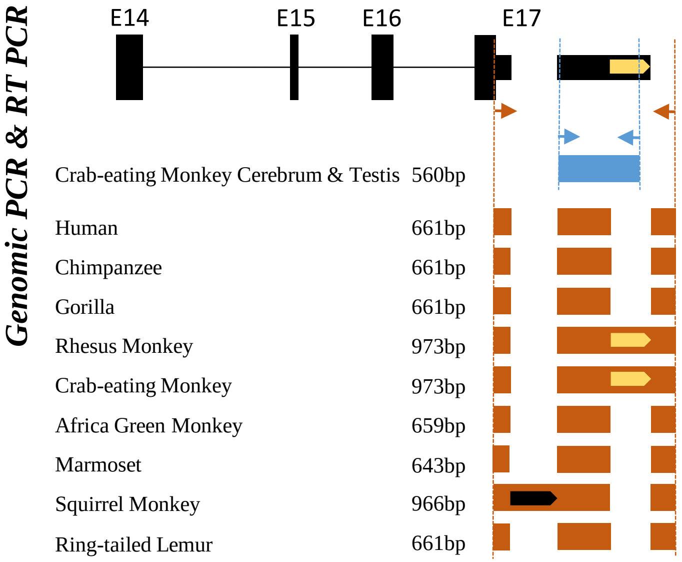

Gel electrophoresis of Genomic PCR products

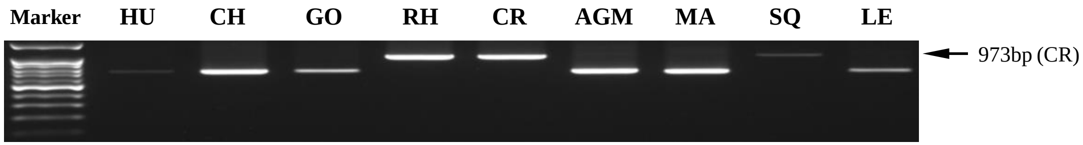

Sequence alignment of Genomic PCR products

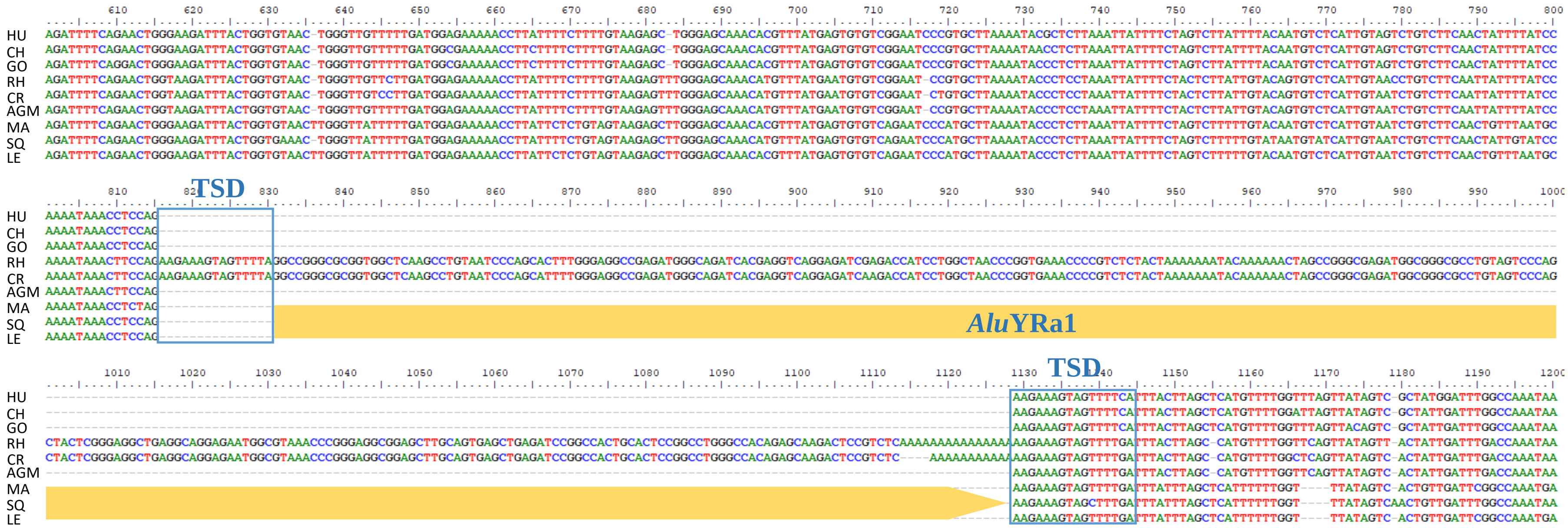

Supplementary Figure S2.

PEX26

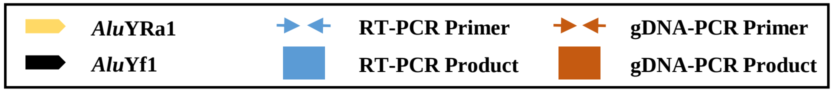

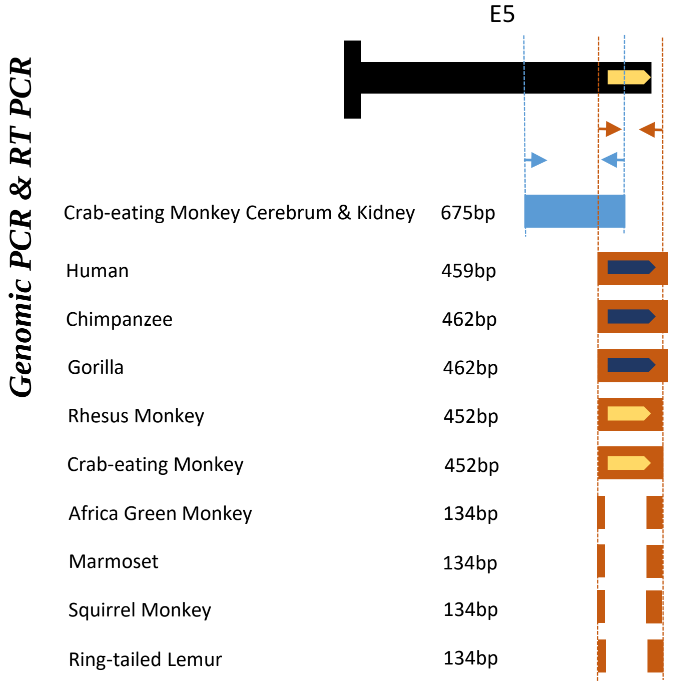

Gel electrophoresis of Genomic PCR products

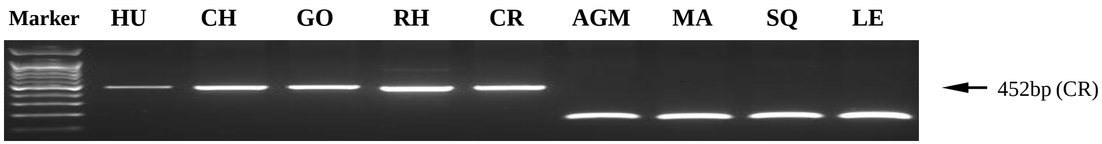

Sequence alignment of Genomic PCR products

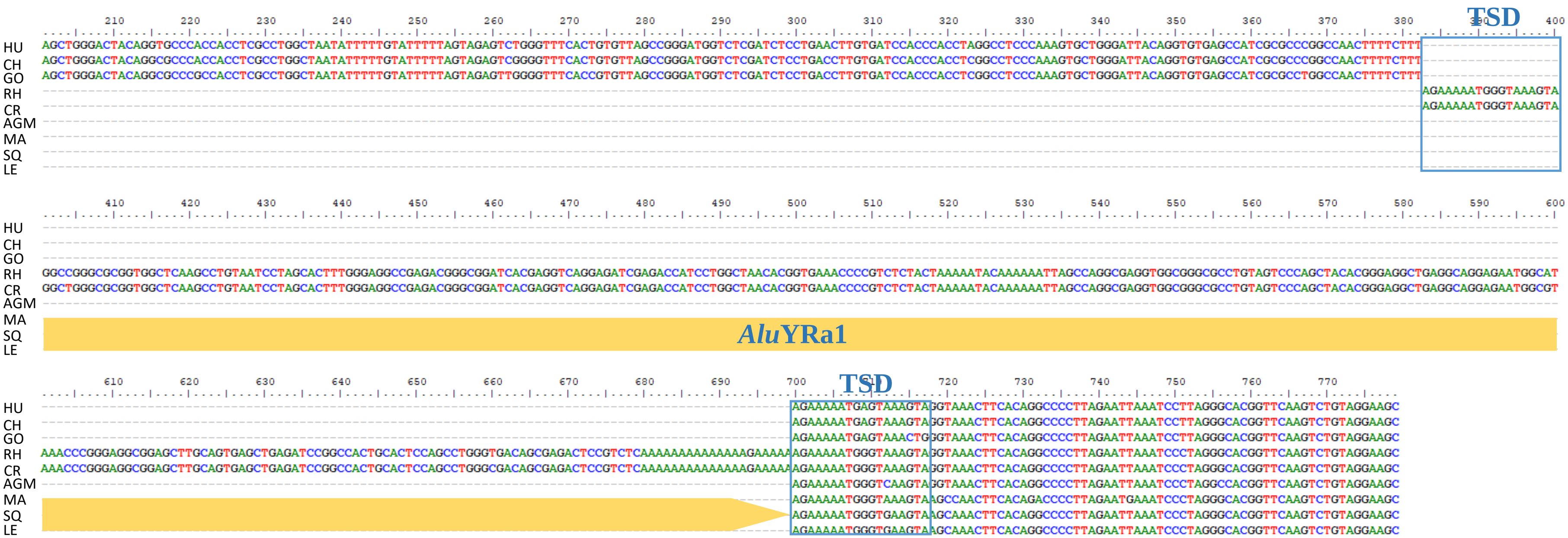

Supplementary Figure S2.

CMBL

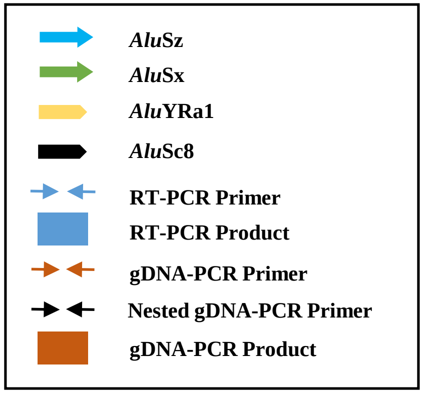

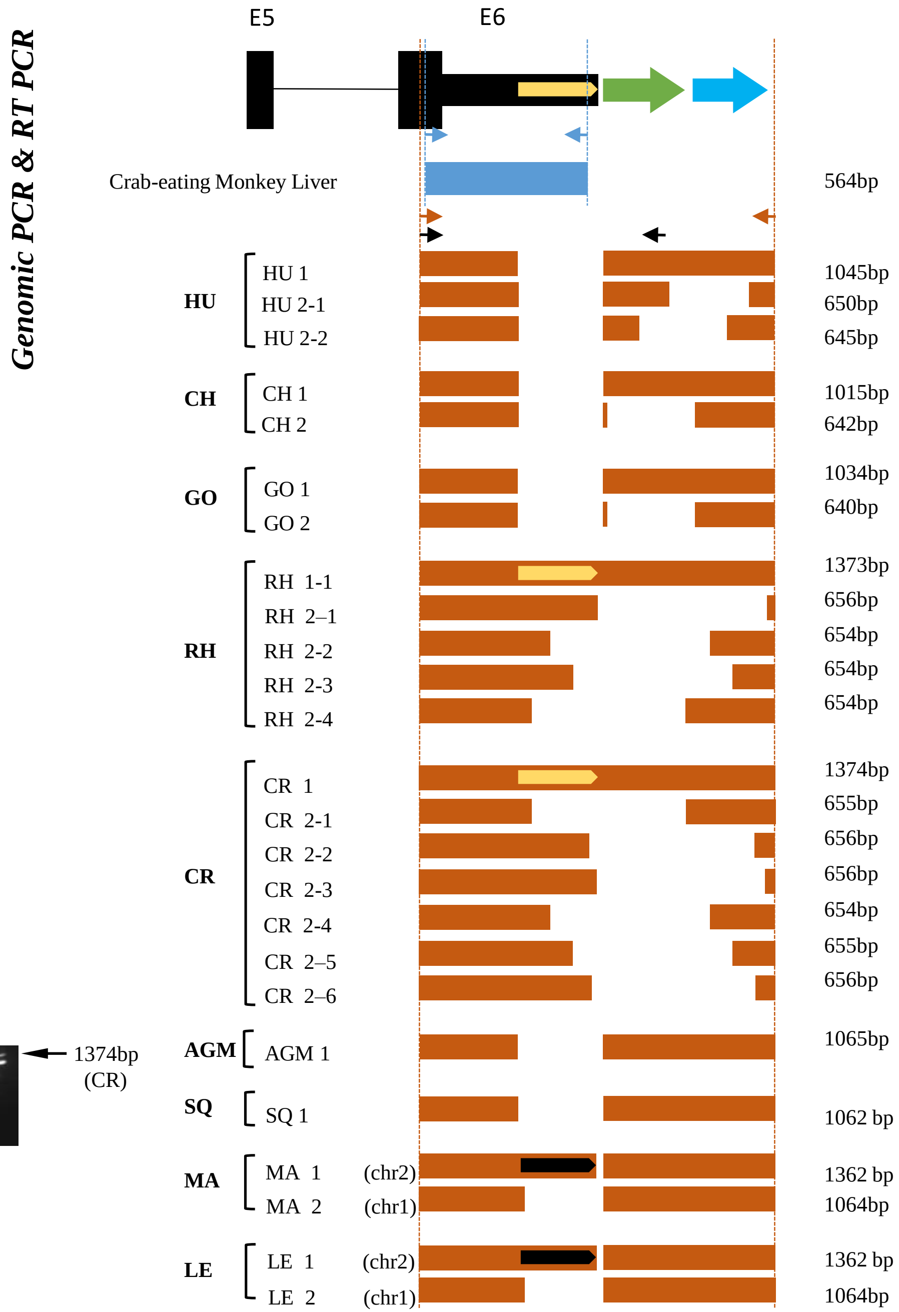

Gel electrophoresis of Genomic PCR products

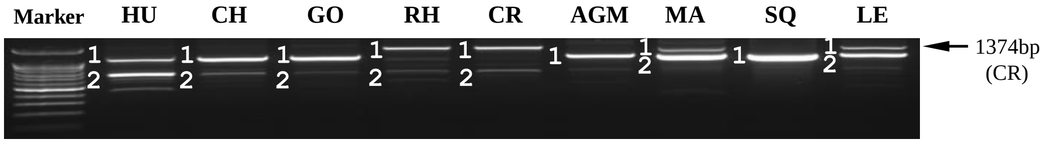

Supplementary Figure S2.

CMBL *Sequence alignment of Genomic PCR products*

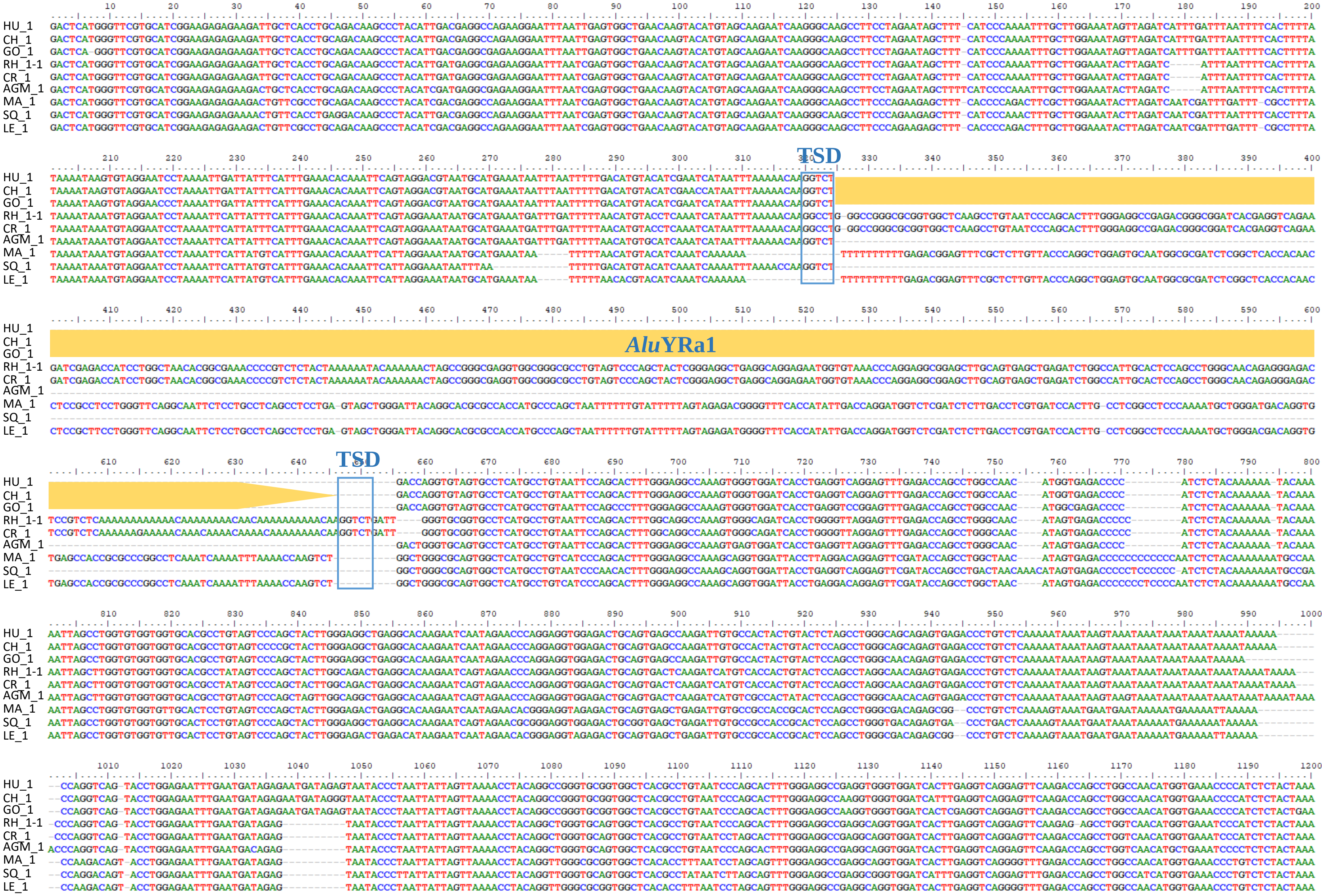

Supplementary Figure S2.

SLC16A14

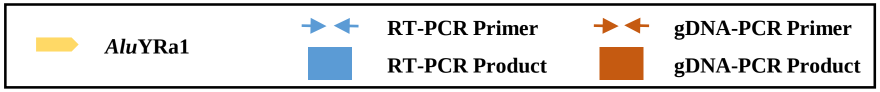

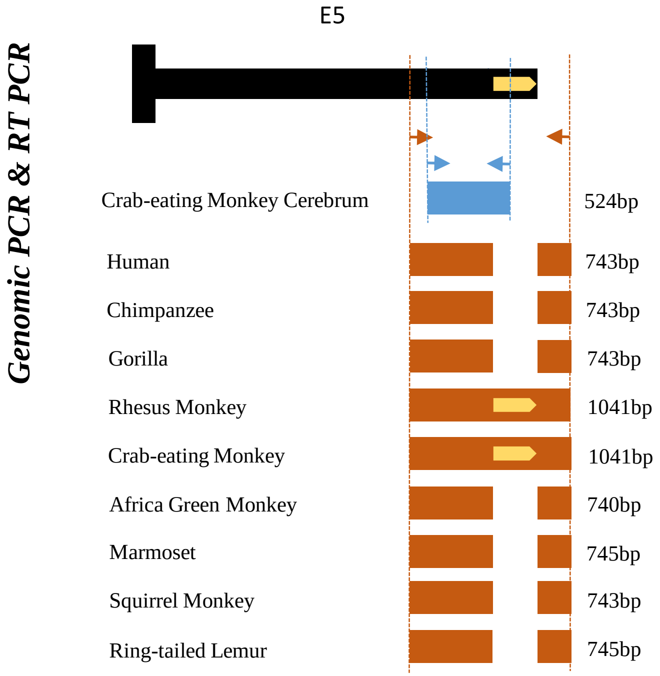

Gel electrophoresis of Genomic PCR products

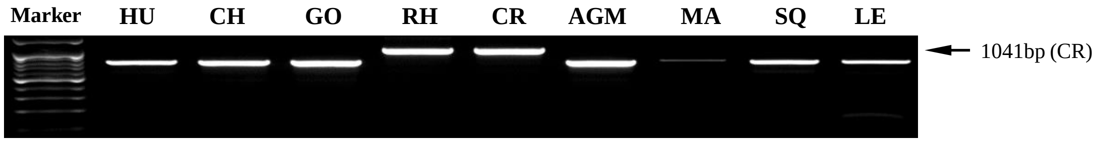

Sequence alignment of Genomic PCR products

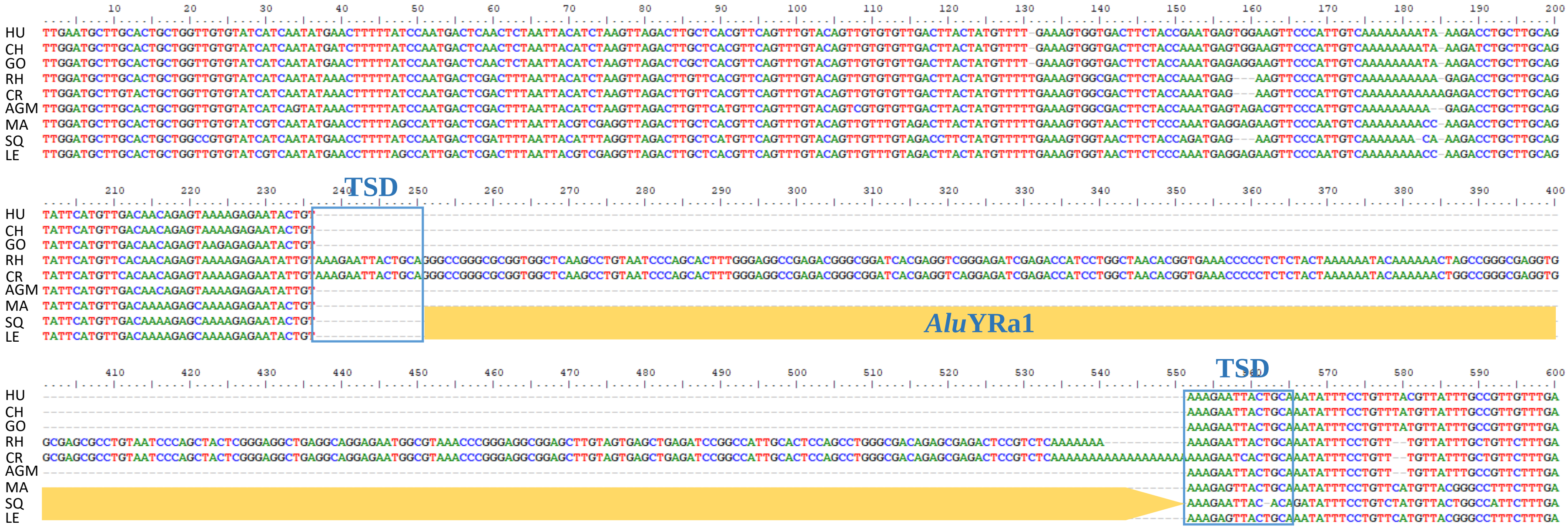

Supplementary Figure S2.

IRF9

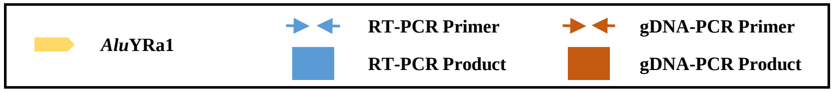

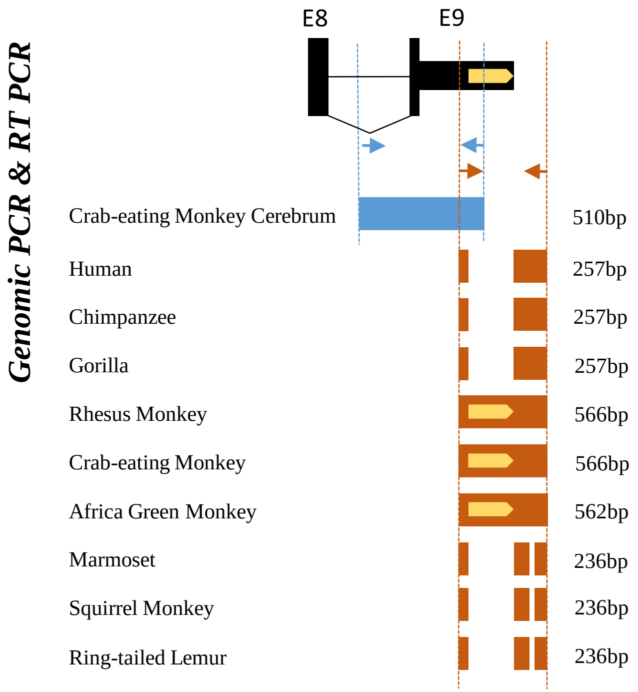

Gel electrophoresis of Genomic PCR products

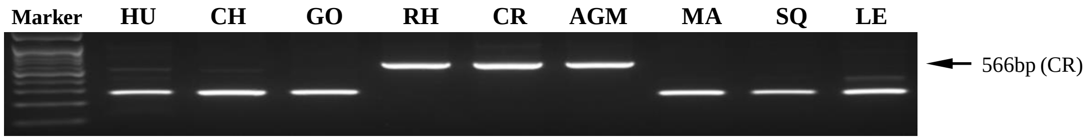

Sequence alignment of Genomic PCR products

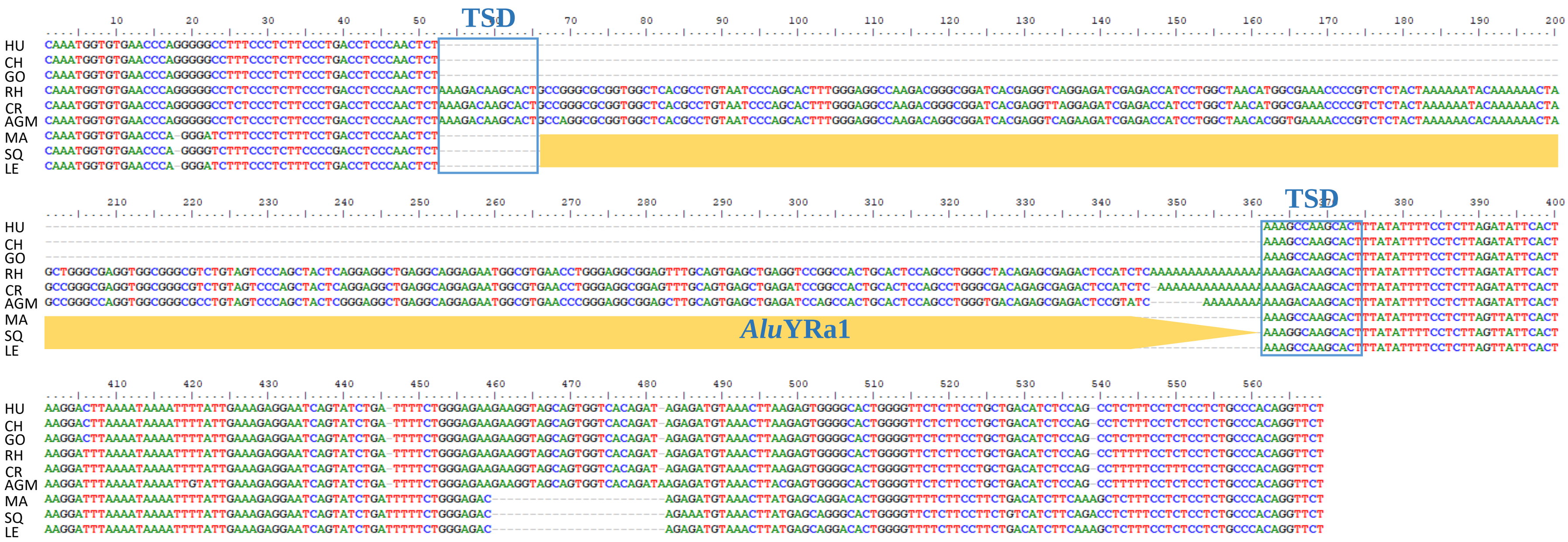

Supplementary Figure S2.

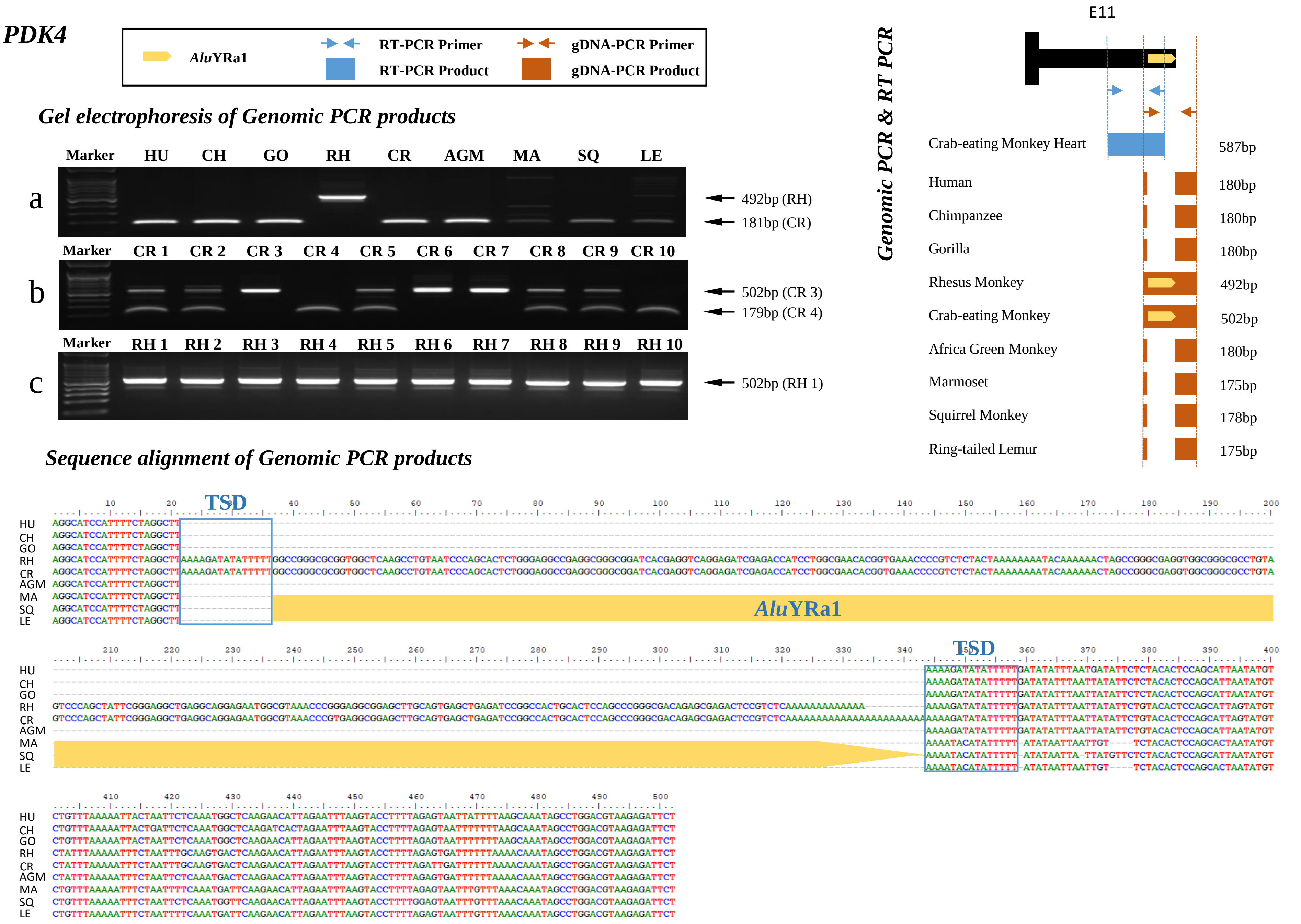

Supplementary Figure S2.

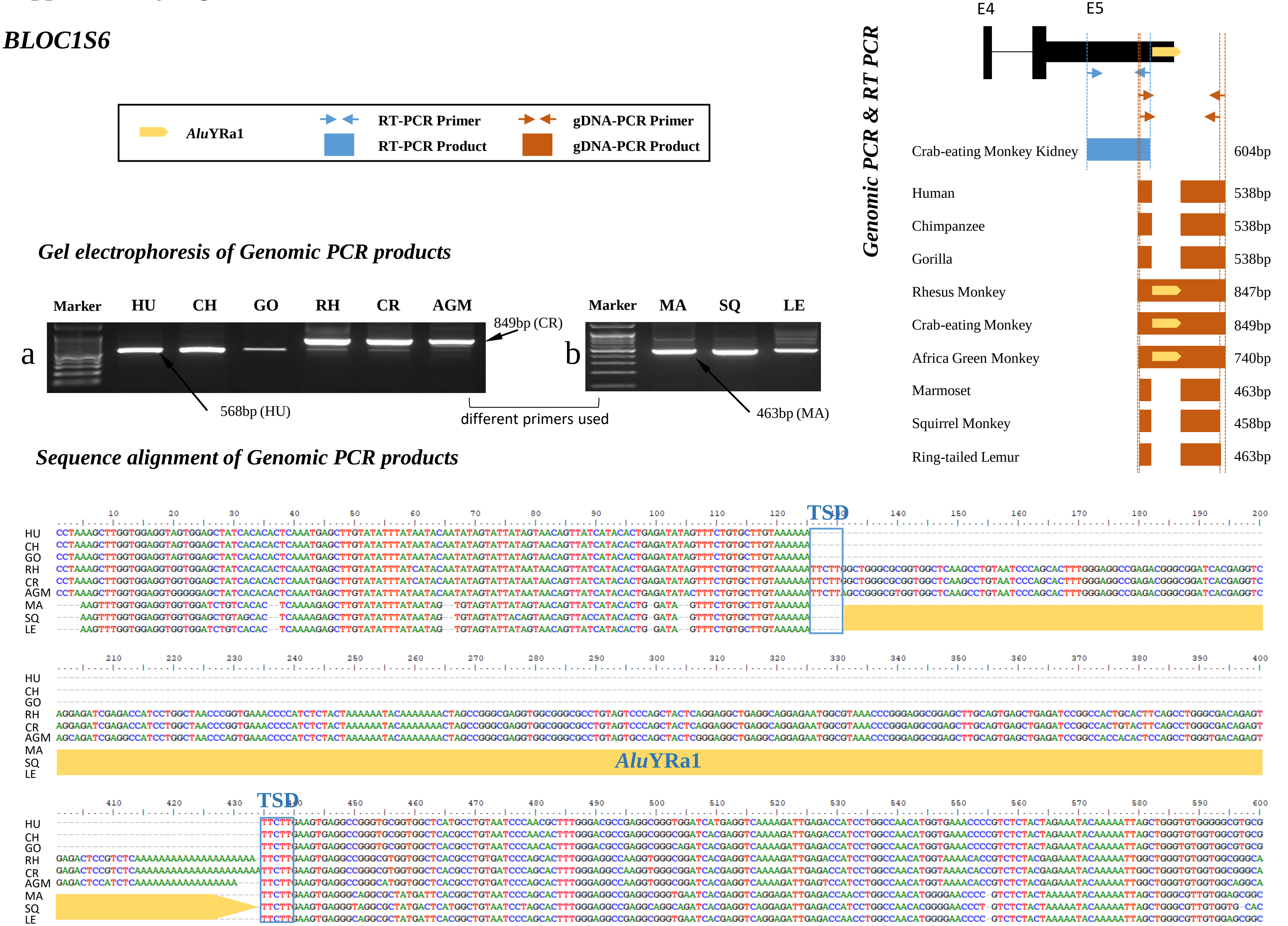

Supplementary Figure S2.

UBE2B

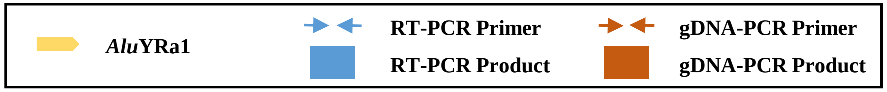

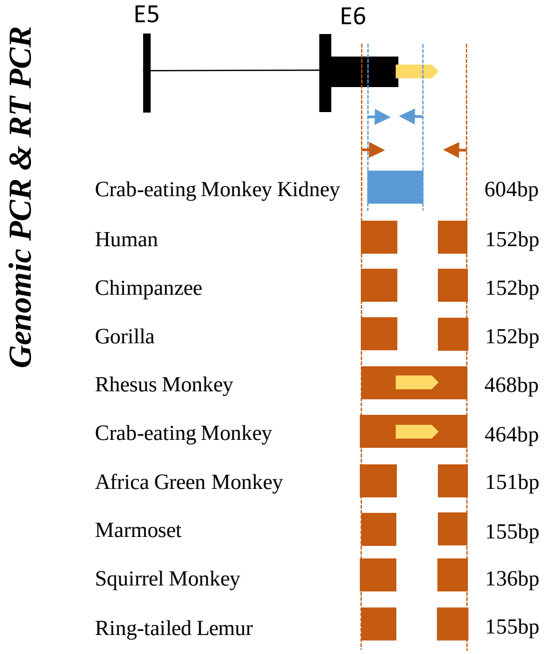

Gel electrophoresis of Genomic PCR products

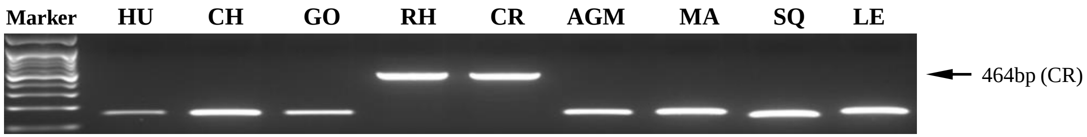

Sequence alignment of Genomic PCR products

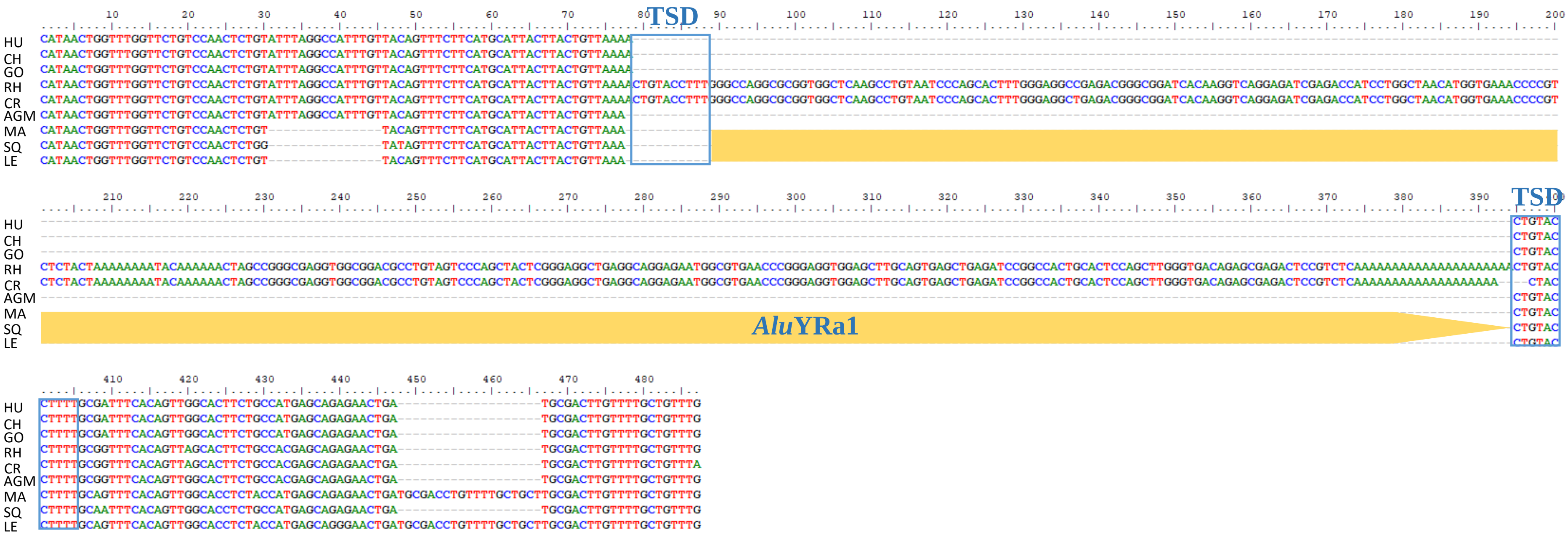

Supplementary Figure S2.

PAICS

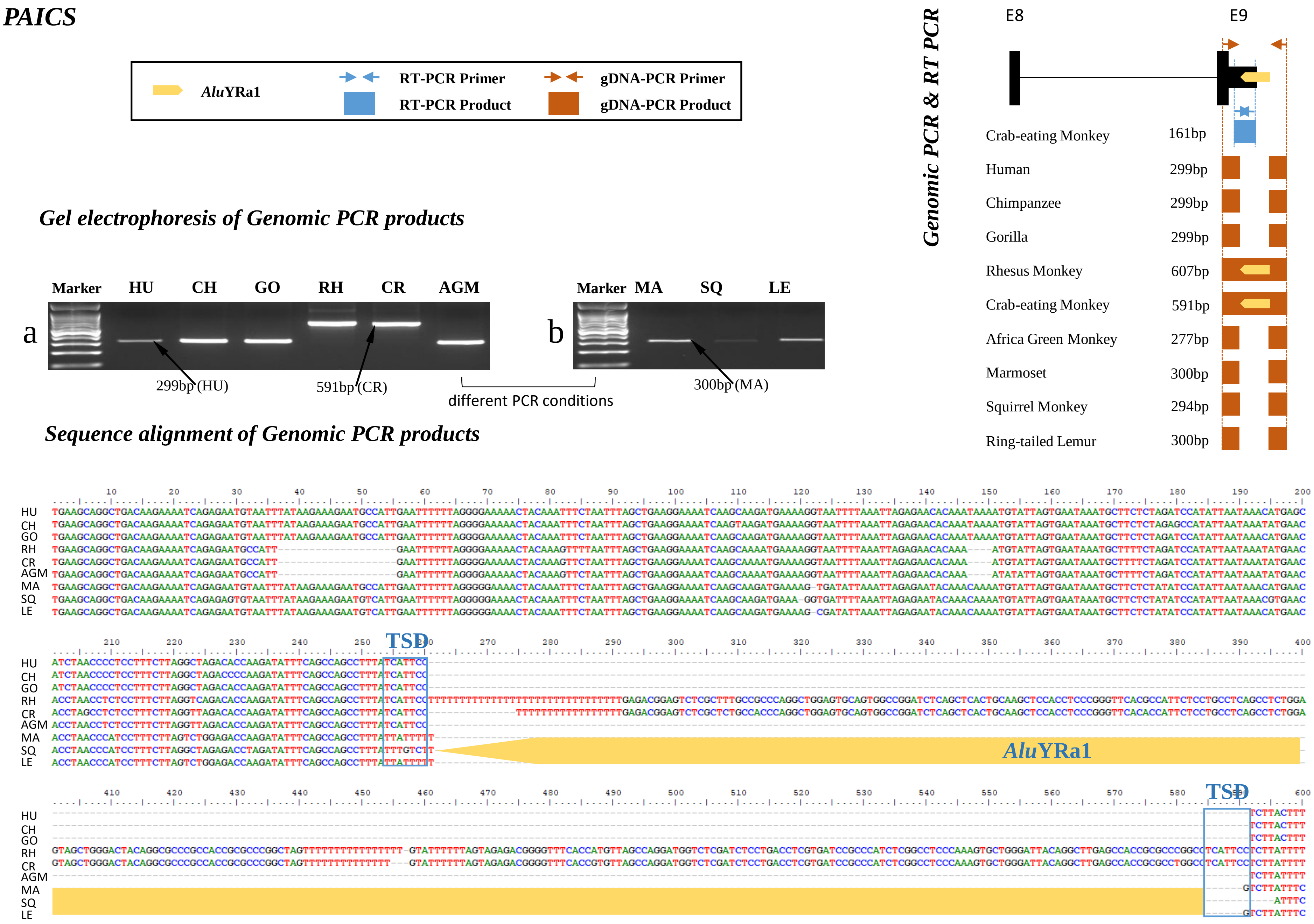

Supplementary Figure S3.

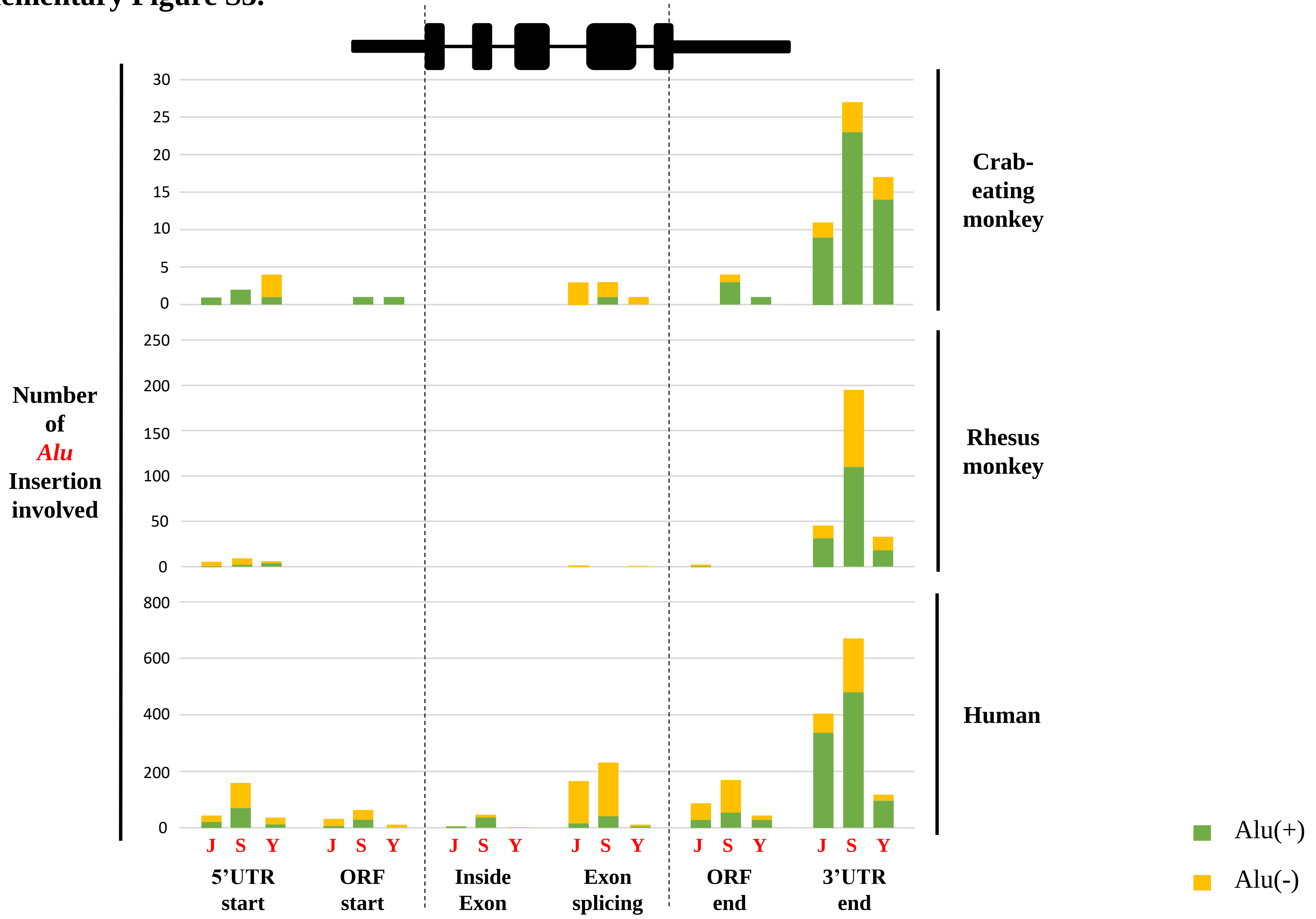

Supplementary Figure S3. Fig. 5a analysis was expanded to classify three *Alu* subfamilies; *Alu*J, *Alu*S, and *Alu*Y.

Green and yellow bars represent inserted repeat elements in sense and antisense orientation against gene direction, respectively.

Supplementary Figure S4.

```
>NM_001283708   PSMA2   81056845        81066231        chr10   +
2intron (TATT)n Simple_repeat   Simple_repeat   81057527        81057556        +       intron
2intron AluYRa1 SINE    Alu     81057556        81057823        -       intron
2intron AluSx1  SINE    Alu     81057850        81058146        +       intron
3intron LTR10B1 LTR     ERV1    81059540        81059939        -       intron
3intron HERVI-int       LTR     ERV1    81059958        81060497        -       intron
3intron AluSx3  SINE    Alu     81060497        81060794        -       intron
3intron HERVI-int       LTR     ERV1    81060794        81061571        -       intron
3intron HERVI-int       LTR     ERV1    81061663        81062249        -       intron
3intron HERVIP10B3-int  LTR     ERV1    81062255        81062698        -       intron
3intron AluSg4  SINE    Alu     81062698        81062999        -       intron
3intron HERVIP10B3-int  LTR     ERV1    81062999        81064312        -       intron
3intron LTR10B1 LTR     ERV1    81064370        81064568        -       intron
3intron AluJo   SINE    Alu     81065386        81065574        -       intron
>NM_001283747   MED20   128735898       128752125       chr4    +
1intron AluSx   SINE    Alu     128736236       128736457       -       intron
1intron AluYRa2 SINE    Alu     128736483       128736793       -       intron
1intron AluSp   SINE    Alu     128737012       128737324       -       intron
1intron FLAM_C  SINE    Alu     128737919       128738060       -       intron
1intron MIR     SINE    MIR     128738428       128738553       +       intron
1intron AluSq   SINE    Alu     128738553       128738858       -       intron
1intron MIR     SINE    MIR     128738858       128738966       +       intron
1intron (TGTTT)n        Simple_repeat   Simple_repeat   128739046       128739083       +       intron
1intron MER3    DNA     hAT-Charlie     128739155       128739240       +       intron
1intron MER113  DNA     hAT-Charlie     128739267       128739419       -       intron
1intron HY1     scRNA   scRNA   128739542       128739654       +       intron
1intron AluSg   SINE    Alu     128739654       128739952       -       intron
2intron AluYRb3 SINE    Alu     128740382       128740670       -       intron
2intron (TTTAT)n        Simple_repeat   Simple_repeat   128740760       128740802       +       intron
2intron AluJr   SINE    Alu     128740803       128741104       -       intron
2intron L1MA10  LINE    L1      128741107       128741274       -       intron
2intron AluJr   SINE    Alu     128741278       128741535       -       intron
2intron AluSx   SINE    Alu     128741537       128741619       -       intron
2intron MER5A   DNA     hAT-Charlie     128741668       128741834       +       intron
2intron AluJr   SINE    Alu     128742043       128742147       -       intron
2intron MIRc    SINE    MIR     128742218       128742333       +       intron
2intron LTR33A  LTR     ERVL    128742340       128742755       +       intron
2intron MIR     SINE    MIR     128742764       128742869       +       intron
2intron AluSq   SINE    Alu     128742870       128743166       -       intron
2intron L2c     LINE    L2      128743384       128743427       -       intron
2intron MIRc    SINE    MIR     128743440       128743528       +       intron
2intron MER5A   DNA     hAT-Charlie     128743537       128743632       -       intron
2intron AluJo   SINE    Alu     128743632       128743887       -       intron
2intron MER5A   DNA     hAT-Charlie     128743887       128743957       -       intron
2intron MIRc    SINE    MIR     128743960       128744026       +       intron
2intron AluSx1  SINE    Alu     128744069       128744369       +       intron
2intron AluJo   SINE    Alu     128744381       128744515       +       intron
2intron AluY    SINE    Alu     128744515       128744812       +       intron
2intron AluJo   SINE    Alu     128744812       128744975       +       intron
2intron AluY    SINE    Alu     128745429       128745802       -       intron
2intron AluSz   SINE    Alu     128745980       128746293       -       intron
2intron L1ME3F  LINE    L1      128746740       128746860       -       intron
2intron AluY    SINE    Alu     128746860       128747130       +       intron
3intron AluJb   SINE    Alu     128747743       128747906       -       intron
3intron AluYRa1 SINE    Alu     128747906       128748207       -       intron
3intron AluJb   SINE    Alu     128748207       128748347       -       intron
3intron (GT)n   Simple_repeat   Simple_repeat   128748822       128748839       +       intron
4exon   MLT1J   LTR     ERVL-MaLR       128751070       128751221       -       3UTR,exon,exonInside
4exon   L4_B_Mam        LINE    RTE-X   128751739       128751780       +       3UTR,exon,exonInside
4exon   Tigger8 DNA     TcMar-Tigger    128752024       128752129       -       3UTR,UTRend,exon,tail
```

output format

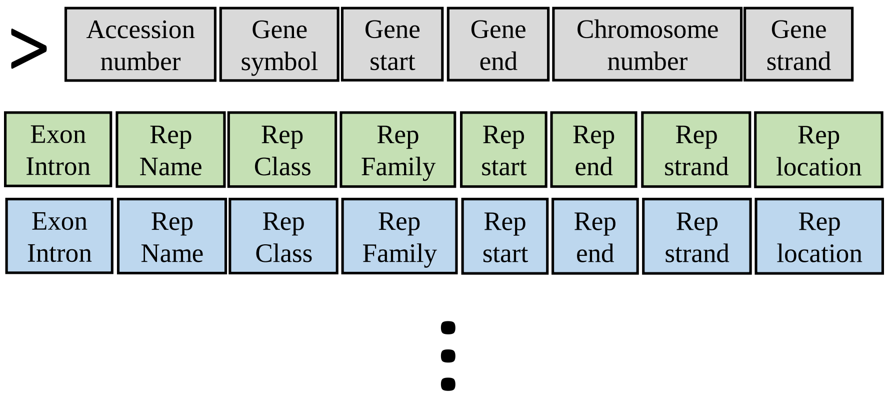

Repeat sequence location in the gene

1. **Whole gene** : repetitive covers whole gene
2. **Intron**: repetitive located at Intron region
3. **5UTR**: located at 5’UTR
4. **3UTR** : located at 3’UTR
5. **UTRcovered** : covers UTR
6. **UTRstart** : located at UTR start position
7. **UTRend** : located at UTR end position
8. **ORF** : located at ORF region
9. **ORFstart** : located at ORF start position
10. **ORFend** : located at ORF end position
11. **m2exon** : located at more than 2 exons
12. **exon** : located at exon region
13. **exoncovered** : covers whole exon
14. **head** : located at exon head position
15. **tail** : located at exon tail position
16. **exonInside** : located inside exon

Supplementary Figure S4. The output file format of the self-devised locator.

Information on the repeat sequence location. The locator was designed by basic Python coding. The output file consists of 14 columns: gene accession number, gene symbol, gene start position, gene end position, chromosome number, gene strand, exon or intron number, repeat name, repeat class, repeat family, repeat start position, repeat end position, repeat strand, repeat location.

Supplementary Figure S5.

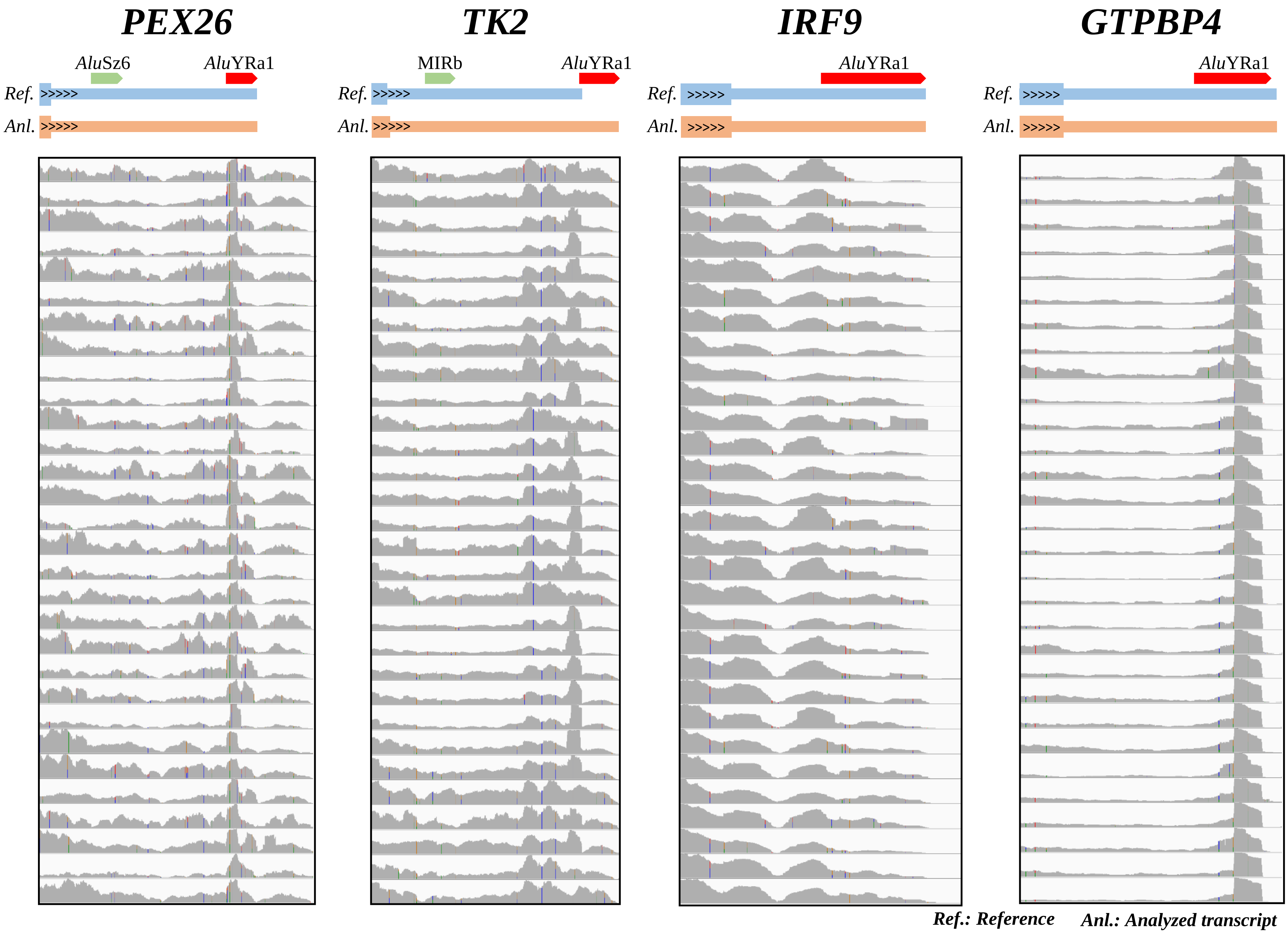

Supplementary Figure S5. 3'UTR read coverage of 30 crab-eating monkey RNA samples on the 4 genes

Supplementary Figure S6.

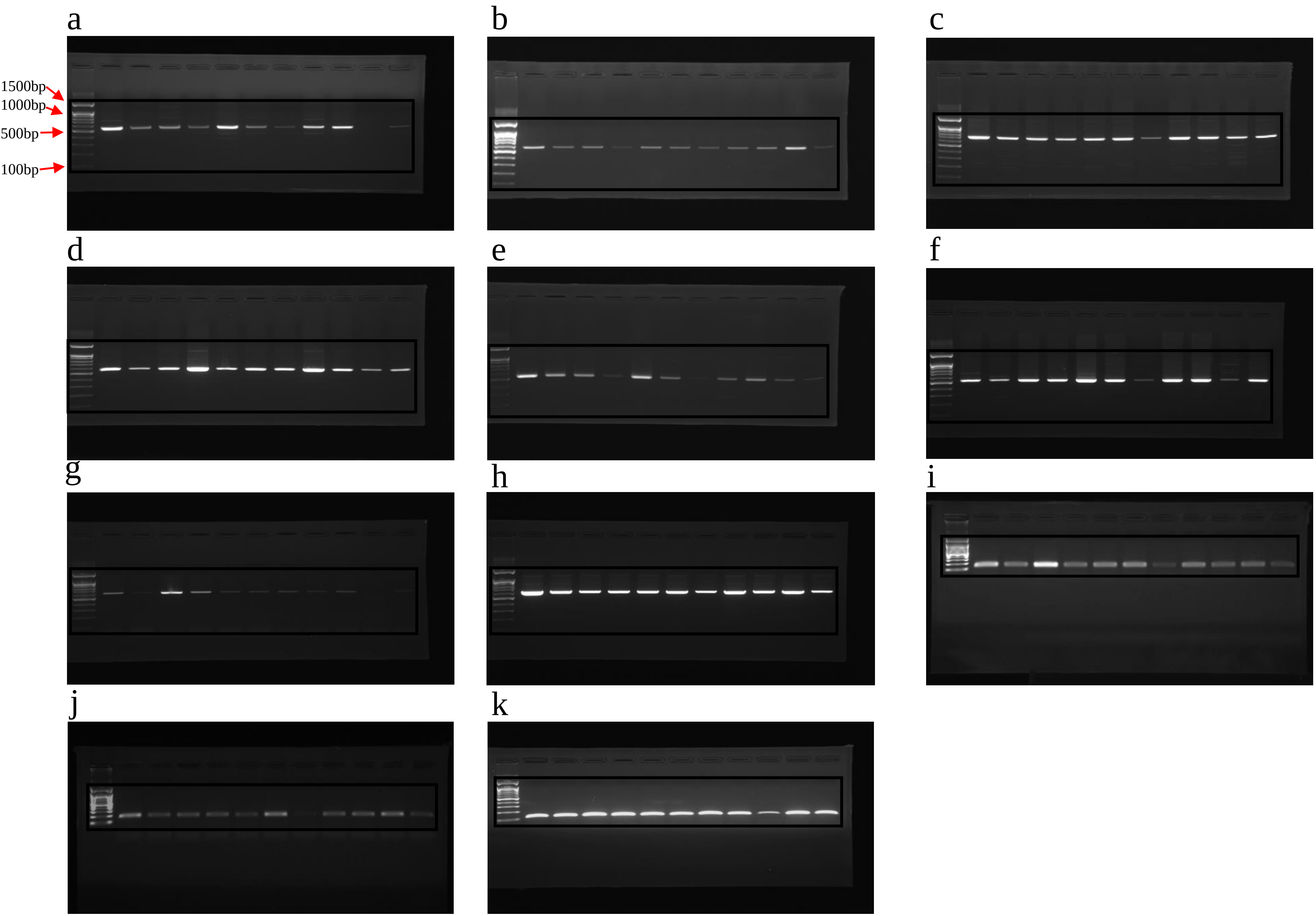

Supplementary Figure S6.

Supplementary Figure S6. Full length of cropped gel images in Figure 4.

100bp DNA ladder is used for the Marker. Solid black lines are cut edge of the cropped gel images in the main Figure 4.

a, *TK2*; b, *GTPBP4*; c, *PEX26*; d, *CMBL*; e, *SLC16A14*; f, *IRF9*; g, *PDK4*; h, *BLOC1S6*; i, *UBE2B*; j, *PAICS*; k, *GAPDH*

Supplementary Figure S7.

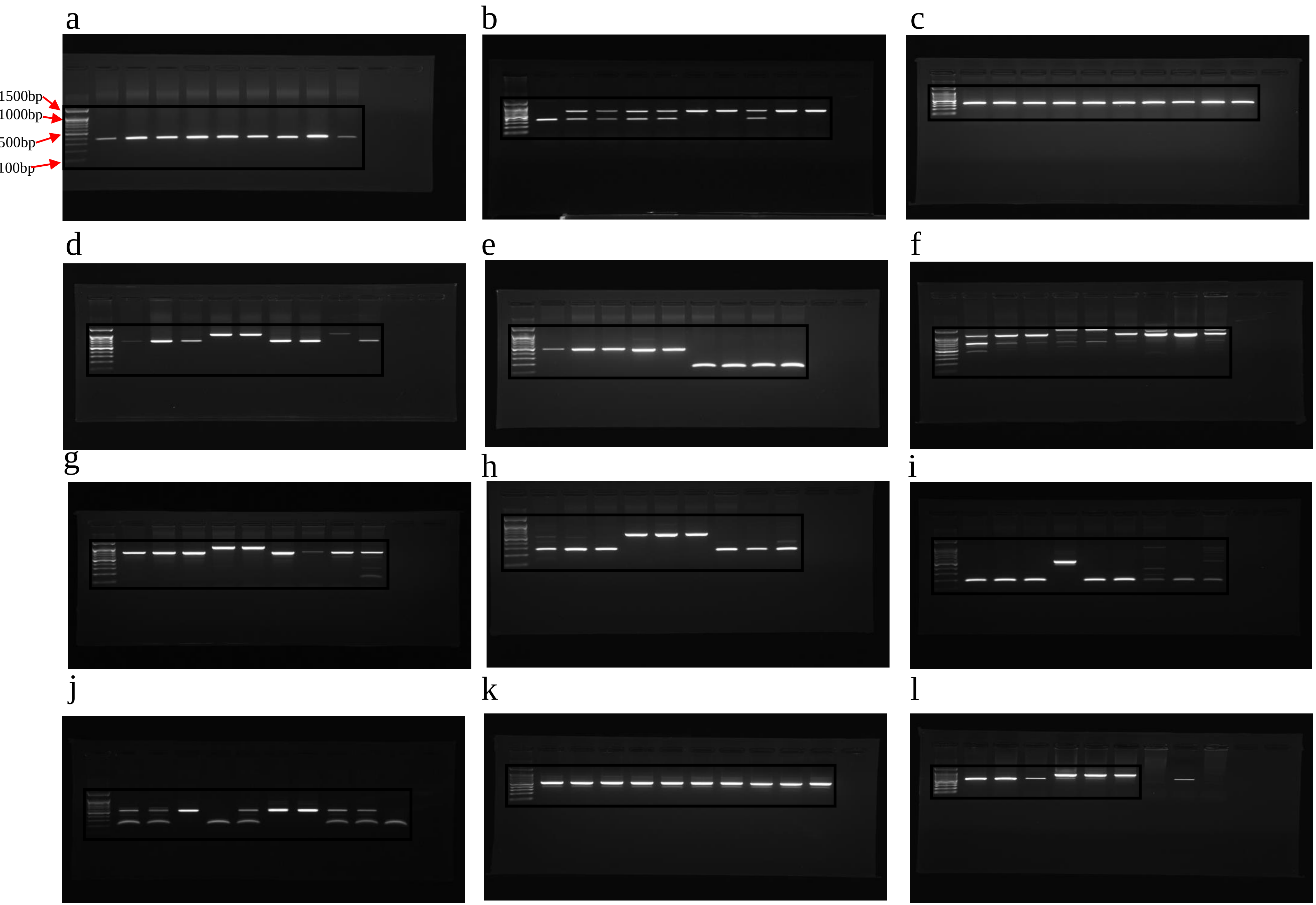

Supplementary Figure S7.

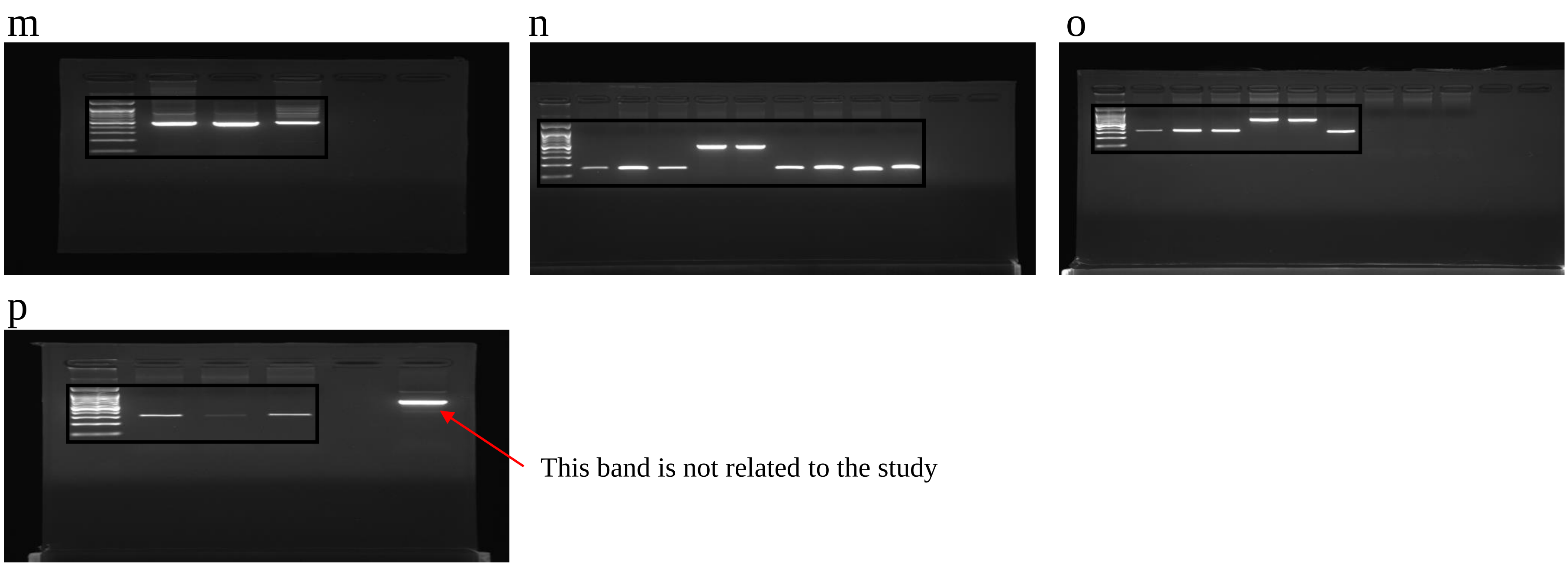

Supplementary Figure S7. Full length of cropped gel images in Supplementary Figure S2.

100bp DNA ladder is used for the Marker. Solid black lines are cut edge of the cropped gel images in Supplementary Figure S2.

a, *TK2*_a; b, *TK2*_b; c, *TK2*_c; d, *GTPBP4*; e, *PEX26*; f, *CMBL*; g, *SLC16A14*; h, *IRF9*; i, *PDK4*_a; j, *PDK4*_b; k, *PDK4*_c; l, *BLOC1S6*_a; m, *BLOC1S6*_b; n, *UBE2B*; o, *PAICS*_a; p, *PAICS*_b
